# Adaptive user interface design and analysis using emotion recognition through facial expressions and body posture from an RGB-D sensor

**DOI:** 10.1371/journal.pone.0235908

**Published:** 2020-07-16

**Authors:** Selma Medjden, Naveed Ahmed, Mohammed Lataifeh

**Affiliations:** Department of Computer Science, University of Sharjah, Sharjah, UAE; National Institutes of Health, UNITED STATES

## Abstract

This work presents the design and analysis of an Adaptive User Interface (AUI) for a desktop application that uses a novel solution for the recognition of the emotional state of a user through both facial expressions and body posture from an RGB-D sensor. Six basic emotions are recognized through facial expressions in addition to the physiological state, which is recognized through the body posture. The facial expressions and body posture are acquired in real-time from a Kinect sensor. A scoring system is used to improve recognition by minimizing the confusion between the different emotions. The implemented solution achieves an accuracy rate of above 90%. The recognized emotion is then used to derive an Automatic AUI where the user can use speech commands to modify the User Interface (UI) automatically. A comprehensive user study is performed to compare the usability of an Automatic, Manual, and a Hybrid AUI. The AUIs are evaluated in terms of their efficiency, effectiveness, productivity, and error safety. Additionally, a comprehensive analysis is performed to evaluate the results from the viewpoint of different genders and age groups. Results show that the hybrid adaptation improves usability in terms of productivity and efficiency. Finally, a combination of both automatic and hybrid AUIs result in significantly positive user experience compared to the manual adaptation.

## Introduction

Human Computer Interaction (HCI) is one of the active fields in computer science. The main research in HCI revolves around providing means of communication between humans and computers through UI. Knowing that humans and computers do not share the same language, the UI should be designed with a concise understanding of its users as well as their needs. The design process of a UI is carried out with an objective to minimize users’ invested efforts during the interaction, both in providing the input and understanding the output. This optimization is reflected through the usability of the UI and the quality of the user experience (UX) during the interaction.

Most software interfaces provide a rigid UI experience where the UI forces users to adapt rather than letting them customize the interface according to their preferences. To some extent, some UIs can be adaptable but not adaptive. An adaptable interface allows the user to change the elements of the UI manually, thus the UI does not adapt autonomously rather permits the adaptation through user’s interaction. This results in an extra effort in terms of the interaction for the user. An AUI on the other hand, automatically adjusts the elements of the UI based on different sets of criteria. An AUI ensures that the elements of the UI will be adapted unobtrusively with minimal user intervention to provide effective and efficient usability and UX.

In recent years, several methods in HCI were proposed to design an AUI that considers the system environment [1, 2] or the users’ personal characteristics. Even though adapting a UI for different devices and environments is a practical requirement, a more compelling reason to adapt a UI depends on the personal traits of the user. Cultural background [3], knowledge level [4], learning style [5], and frequency of performed tasks [6] have been explored in the previous research to adapt a UI.

An AUI that relies on the previously mentioned user characteristics results in better usability and positive UX. This suggests that a single static interface is hardly fit for the preferences of all its users. Hence, to achieve higher levels of UX, it is crucial to adapt the interface to each user separately. Identification of the different levels of human traits required for the adaptation, along with the maximum possible degree of adaptation are the main challenges of the design of an AUI [7, 8]. Although these studies proved the positive effect of adaptability on usability goals, enhancing the UX through adaptability is still an open challenge.

An emotion can be defined as a mental state associated with the changes that happen internally (chemical and physiological signals) and expressed externally (facial expression, body posture, movements, and speech utterance) as a response to some incident [9]. Emotion recognition relies on these changes to automatically assess the emotional state of a person. In 1970, Ekman and Friesen [10] stated in one of their research that universally, humans express six basic emotions: happiness, sadness, anger, fear, surprise, and disgust.

Ekman and Fiesen [10] designed a facial action coding system (FACS). This system provides a mapping between the six basic emotions [11] (happiness, sadness, anger, fear, surprise, and disgust) and all possible facial muscle movements. These muscle movements are coded in terms of 44 action units (AUs). Each of the six basic emotions is linked to one or more AU. A typical facial expression emotion recognition solution involves the following steps: detection, tracking, feature extraction, and classification.

A number of methods have been employed for face detection, namely, Haar classifiers [12], adaptive skin color models [13], and AdaBoost [14]. For the face tracking, features are first tracked from each frame then tracked over a sequence of frames. The feature extraction phase consists of identifying the facial features that will be used in the classification phase. The choice of features is generally influenced by the classification technique. Most of the feature extraction and tracking techniques are Geometric based [15–19].

The classification phase generally uses a machine learning algorithm that takes the extracted feature vector as an input and outputs the resulting emotion. Support Vector Machines (SVM) [20], Neural Networks (NN), and Hidden Markov Models (HMM) are the most common classification algorithms. Other than facial expressions, some facial features have been explored in the context of emotion recognition, for examples eye-based features such as pupil diameter [21] and eye gaze [22].

The use of body posture as a feature to recognize human emotion is a topic that was debated through the literature. McNeill [23], argues that the hand gestures are just a secondary mean of communication that only accompanies the speech modality. Some researches even stated that body posture does not add any information about human emotions on their own [9]. On the other hand, humans naturally express themselves using more than one modality including body posture [9, 24]. Hence, more researchers have taken an interest in exploring the use of body posture to recognize emotions. Moreover, Calvo et al. [25], stated that body postures are harder to control than facial expression which implies that body postures might result in more genuine recognition. Emotion recognition using body posture is comparable to facial expression recognition. It involves the detection and tracking of the human body, body features extraction and finally classification [26]. Multiple approaches that show body cues alone and or a combination of body cues and facial expressions are more reliable to perceive the negative and positive emotions as presented by Aviezer et al. [27] and Martinez et al. [28]. They demonstrate that people can perceive the emotions either directly through the posture without looking at the face, e.g. a celebrating player, or a combination of both facial and body cues.

In this work, a Kinect v2 sensor was used to acquire the input frames. Kinect v2 provides color, depth, and pose data of a person at 30 frames per second. In addition, Microsoft provides an SDK that includes a face tracking API. HD Face API allows the detection and tracking in real-time of more than 1000 facial points. It also provides a total of 17 action units (AUs) that are based on the Facial Action Coding System (FACS) [10]. Considering the advantages that are provided by the sensor and its SDK, a decision was made to use Kinect v2 and the provided HD Face API for the real-time data acquisition that forms the input of the facial emotion recognition system. The facial data is used to recognize six emotions: happiness, sadness, anger, fear, surprise, and disgust. Additionally, real-time pose data was used for the detection of tiredness or fatigue. There have been some recent works [29–32] that detect emotions using multiple modalities, but so far, to the best of authors’ knowledge, it has not been employed to derive an AUI.

There has been a number of methods proposed to create an AUI that considers various traits of the user. In [4] and [5], based on the learning style of the user, an AUI was designed to present adequate contents. Additionally, the learning style is also acquired to identify whether the user prefers a more general overview of the available resources or be presented with only specific components. In [6], the layout of a professional geographic information system interface was adapted. The strong information presentation aspect of the interface and the characteristics of its user group influenced the choice of the system. To adapt the UI, the authors used a split menu strategy that was introduced in [31]. This strategy roughly relies on rearranging the layout of the menu items based on the frequency of clicks while keeping the traditional alphabetical order. The split menu strategy makes it easier for the user to locate the items they use more frequently by placing them at the top of the menu. In addition, based on [32], suggested menu items were also highlighted, and non-suggested menu items were shrunk. Allowing the user to have a less crowded visual space during the interaction.

A game biofeedback mechanism was implemented by monitoring the breathing rate of the current player by Parnandi et al. [33]. They consider that a high breathing rate during interactions implies that the player is feeling anxious and needs assistance. In [34], to increase the level of engagement of the player/student, Katmada et al. designed an educational game that adapts according to the level of anxiety of the student. Nasoz et al. [35] presented an adaptive car interface. The solution relies on age, gender, personality traits, and emotional state of the driver in order to provide real-time adaptation. AUBUE [36], is an AUI that relies on the user’s emotional state to change the color of the UI background. None of the works in AUI design take into consideration seven different emotions from multiple modalities.

In order to evaluate the performance of a user interface, several techniques have been used in the past. Two types of evaluation techniques stand out. The first type relies on an expert analysis who tries to achieve tasks using the interface as a common user would. This allows the expert to infer the performance of the interface from the users’ perspective. Some techniques that fall under this type of evaluation include a cognitive walkthrough, heuristic evaluation, model-based evaluation, and review-based evaluation. In the second type, the evaluation is performed through user participation. In this type of evaluation, participants are asked to use the interface and provide feedback on their experience. Some of the evaluation techniques that fall under this type include experimental evaluation, observational methods, query techniques, and physiological methods. In this work an experimental evaluation was conducted that will be detailed in Section ‘Experiment Setup and Testing’.

In [6], to assess the effect of the adaptivity on a WebGIS interface, the authors evaluated, effectiveness, efficiency, and UX. The correctness of the task solution was measured by experts to compute the effectiveness. Efficiency on the other hand was computed by measuring the task completion time and cognitive workload. To measure the cognitive workload Raw NASA Task Load Index [37] was used. Finally, UX was evaluated using the AttrakDiff questionnaire [38].

To assess the effect of adaptation on the learning process of students through a learning system, authors in [5] used statistical analysis, which relied on hypotheses. A null hypothesis and an alternative hypothesis are defined. The null hypothesis is meant to be rejected based on statistical measurements computed from the collected data. Once the null hypothesis is rejected, the alternative hypothesis is automatically supported. The null hypothesis implies that no improvement was achieved in the learning process of the students with the adaptation. Consequently, the alternative hypothesis implies the opposite. In order to address the hypotheses, quiz marks that participants performed were used to compute the average values for all participants. The scores of the quiz were divided into two groups. The first group contains the scores before the adaptation and the other group contains the scores after the adaptation. Paired two-tailed t-tests were used to compare the average values of the two groups. A similar analysis was performed in [39], where the effectiveness, efficiency, and difficulty of the tasks were evaluated by comparing the player’s performance with and without the adaptation.

In this work, three different AUIs are designed for a simple desktop application. The Automatic AUI allows the adaptation via the user’s emotions through multiple modalities and offers an active adaption with the context of the task performed via speech based on the emotion, such that the user does not need to manually adapt the system. The complete Manual AUI that allows the user to customize the user interface using manual interactions. And finally, a Hybrid AUI that combines the elements of both Automatic and Manual AUIs. A comprehensive user study is performed using mixed-methods approach to test each AUI and validate its usability and UX both quantitatively and qualitatively. The quantitative measurements allowed the computation of effectiveness, productivity, efficiency, and error safety of the interfaces. These metrics give even more insight on the usability of the interfaces. The hypotheses testing similar to [5] and [39] was employed. The user study involves 60 participants from three different age groups from 18 to 70 years old. The obtained data is evaluated from multiple viewpoints to identify the variations due to gender differences or age groups. The qualitative measurements were obtained through a user satisfaction questionnaire that contains both questions specific to each AUI and comparative questions. To the best of authors’ knowledge, this study is possibly the first in the literature that combines multimodal emotion recognition with an adaptive user interface and performs a comprehensive user study.

The main contributions of this work are:

1. Designed three different AUIs for an everyday (non-mission critical) desktop application: Automatic, Manual, and Hybrid.
  - The Automatic AUI, is a context-aware adaptation engine that suggests UI adaptations based on the emotions of the user. The user can quickly adapt an interface via speech commands.
  - The Manual AUI allows the UI adaptation through a manual interaction. This interface provides an adaptation mechanism that is triggered by user’s interaction.
  - The Hybrid AUI that combines the elements of both Manual and Automatic AUIs.
2. Developed a multimodal human emotion recognition system that uses Kinect v2 to track the facial features of the user and identify six basic emotions using a novel heuristics-based solution. Furthermore, the posture data from Kinect is also used to detect the level of fatigue or tiredness of the user. The recognized emotions are then used to suggest UI adaptation to improve the UX in the Automatic and Hybrids AUIs.
3. Performed a comprehensive user study using a mixed-methods approach to validate the feasibility of each AUI in terms of its usability and UX. The user study considers effectiveness, productivity, efficiency, and error safety to assess the performance of each AUI. Additional to the usability analysis, the data is also analyzed from the viewpoints of both genders and three age groups, comparatively and individually. Finally, a detailed qualitative analysis was also conducted to measure the UX of each AUI.

In the following sections, the methodology is presented in Section ‘Methodology’, afterward the experiment setup is presented in Section ‘Experiment Setup and Testing’, followed by results and analysis in Section ‘Results and Analysis’, and finally the paper concludes in Section ‘Conclusions and Future Work’.

## Methodology

**Fig 1:**
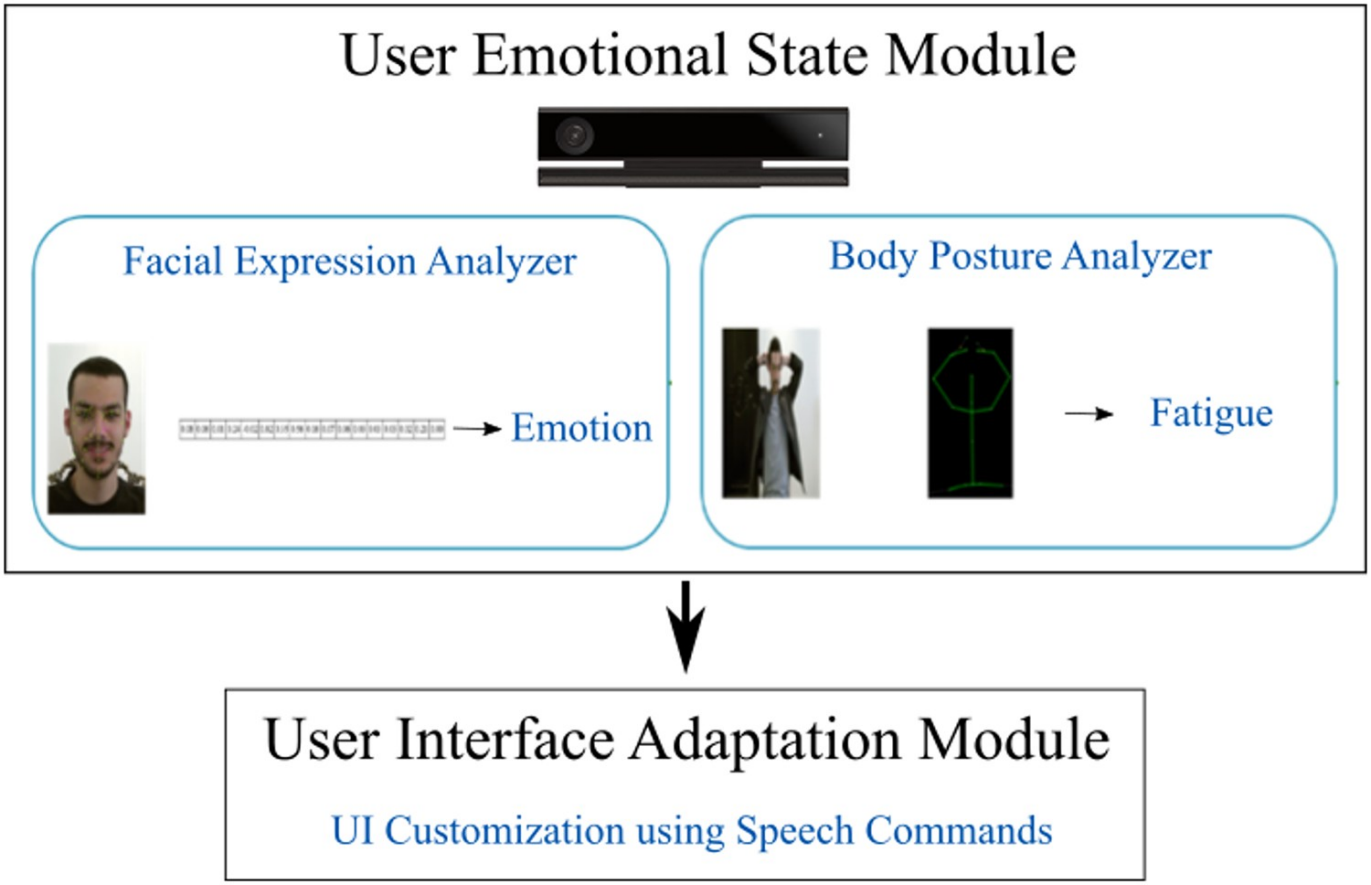
Proposed adaptation framework.

The goal of this work is to assess the impact of adaptation based on emotions expressed during the interaction. For this purpose, a framework was devised to allow the integration of both emotion recognition and UI adaptation. The strategy of this design revolves around different modules exchanging information as illustrated in Fig 1. This ensures a simple integration of other modules or modifications of current ones if necessary. Additionally, a user study was conducted to measure the impact of each emotion-driven adaptation on the usability and the overall UX.

### User emotional state module

This module is designed to recognize 7 emotions from 2 different modalities. It is built from two different components: The Facial Expression Analyzer and the Body Posture Analyzer. The Facial Expression Analyzer is used to recognize Anger, Disgust, Fear, Happiness, Sadness, Surprise, and the neutral state of the user. The Body Posture Analyzer is used to recognize when the user is tired. Both analyzers rely on heuristics that are explained below.

### Facial expression analyzer

In this component, facial features are extracted using Kinect v2 sensor. Kinect v2 along with its SDK, detects and tracks the face at a rate of 30 frames per second and provides a large set of features. The features that this work relies on are Action Units (AUs). A total of 17 AUs are acquired. These AUs are weights that reflect the degree of the movement of the facial muscles compared to the neutral face. Table 1 shows the list of AUs:

**Table 1:** Kinect v2 face tracking SDK action units.

Facial Muscle	AUs
Cheek	Cheek puff (left, right)
Eye	Eye closed (left, right)
Eyebrow	Eyebrow lowerer (left, right)
Jaw	Jaw open, Jaw slide
Lip	Lip pucker, Lip stretcher (left, right), Lip corner puller (left, right), Lip corner depressor (left, right), Lower Lip depressor (left, right)

These features are fed to a new heuristic-based algorithm that outputs the emotional state of the user after going through a scoring system that minimizes the confusion between the different emotions. The Facial Expression Analyzer consists of two main phases, the calibrations phase, and the recognition phase. In the calibration phase, the system saves the facial attributes of the current user for each emotion, this allows the system to fit the recognition to the current user. The recognition phase is where new facial attributes are acquired then processed to output the recognized emotion.

**Fig 2:**
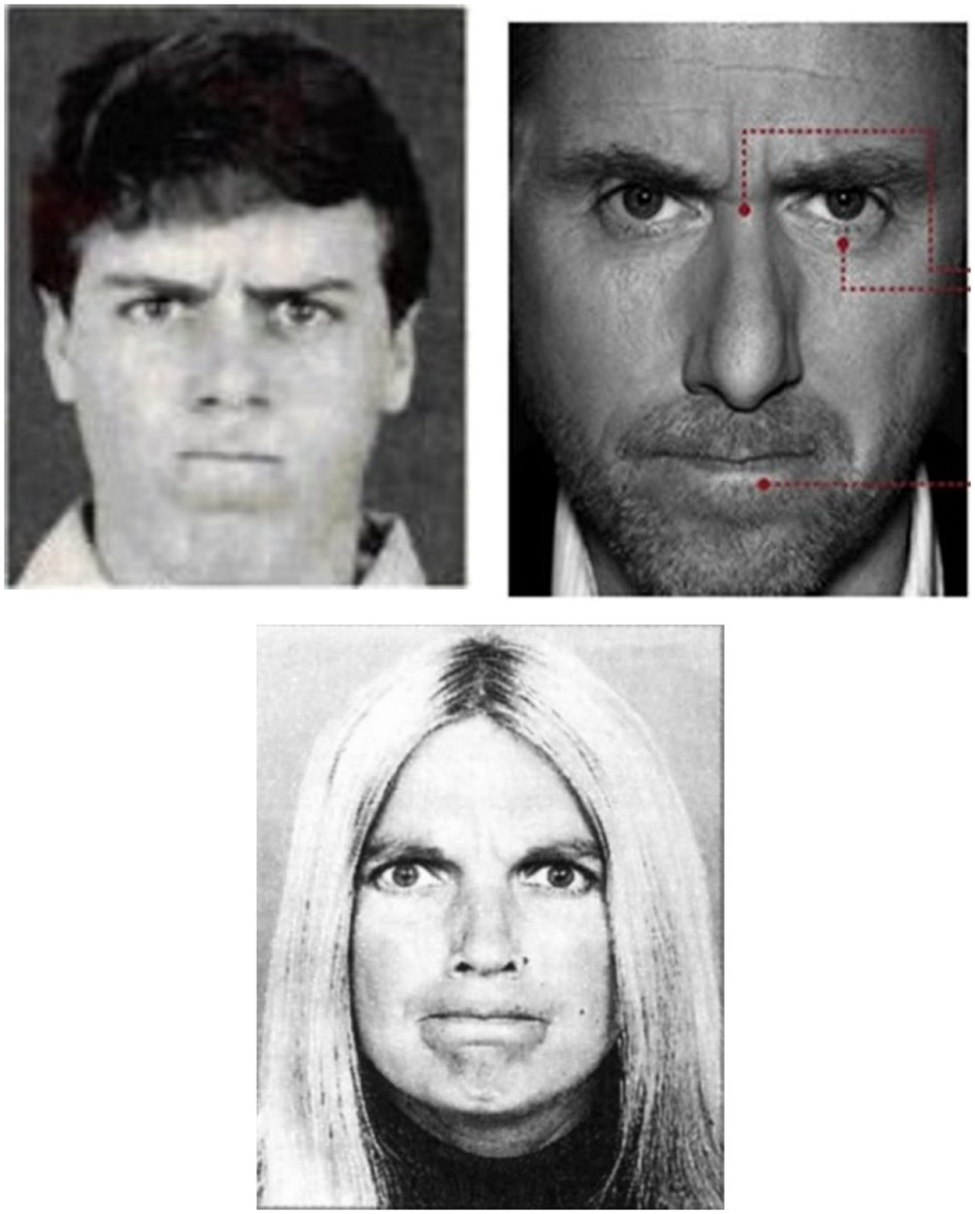
Reference pictures for anger. All other reference images can be seen in the supplementary material (S1 Appendix).

The first phase is to record reference facial attributes to which new data will be compared to during the recognition phase. Through experimenting, it was deduced that having three references for each emotion provides better insight into user’s expression of emotion. A total of 21 reference pictures are used. First the user is presented with a picture of the emotion that needs to be referenced in Fig 2 (all of the reference pictures of emotions can be seen in S1 Appendix). These pictures are meant to trigger the referenced emotion. The user is then asked to mimic the corresponding emotion for approximately 5 seconds (150 frames). Then, the system saves the 17 AUs values representing the facial attributes of the user during the expression of the corresponding emotion.

**Fig 3:**
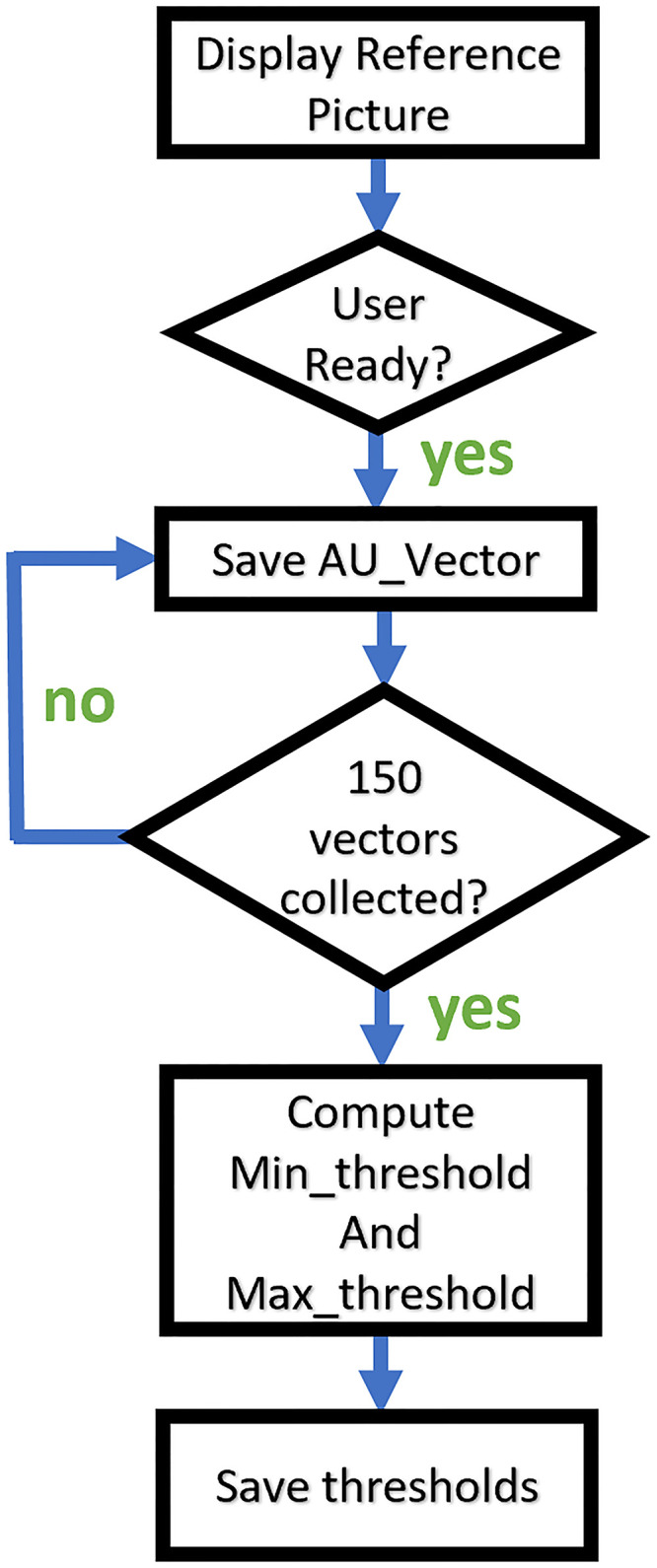
Calibration phase flowchart for one reference picture.

Once the AUs are recorded for the first picture, the minimum and maximum values are computed over the 150 frames. Before saving the minimum and maximum values as thresholds, these values are rounded to the next quarter to normalize the collected data, see Fig 3 for the flowchart. This process is repeated for each emotion, for each triggering picture. At the end of the calibration phase, three minimum threshold vectors, and three maximum threshold vectors will be stored for each emotion. Each vector contains the 17 values of the AUs. Once the calibration phase is completed, the thresholds can be used to classify new AUs feature vectors extracted during the recognition phase.

The recognition phase starts by acquiring new frames from the Kinect sensor. Each frame contains new AUs, representing the facial features of the user at the current time. Next the new AUs are extracted, normalized then saved as a feature vector. The normalization consists of rounding the values to the next quarter. Then, the new values are compared with the minimum and maximum thresholds. The emotion corresponding to the first matching pair of minimum and maximum thresholds is selected as the primary result and the score of this emotion is incremented.

**Fig 4:**
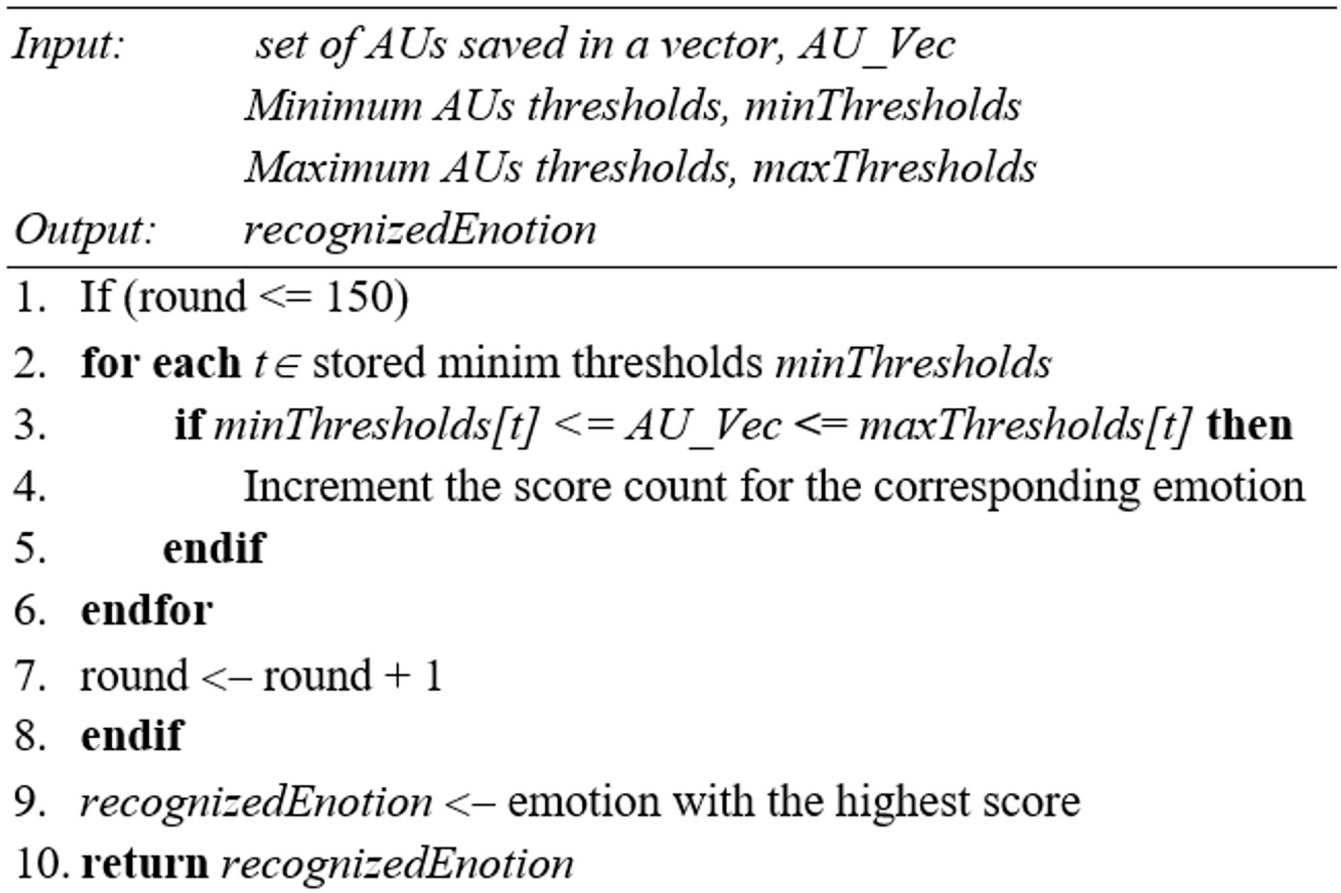
Facial emotion recognition algorithm using AUs.

Finally, the primary result of the recognition phase is passed through a scoring system. The scoring system relies on a system that counts the number of occurrences of each emotion over 150 frames. The emotion that has the highest number of occurrences is selected as the final result. The algorithm in Fig 4 demonstrates the emotion recognition procedure.

### Body posture analyzer

The body Posture Analyzer is used to detect whether the user is tired or not. Prior studies and general knowledge were used to select postures that typically reflect this emotion [24–26]. First the skeleton data is acquired using the Kinect sensor. The sensor provides 25 joints for the full-body skeleton. Considering that the user will be seated during the interaction, this work only relied on the upper body joints in this process. The distances between the upper body joints were used to identify the different body postures. In case the body of the current user is in the predefined posture, the user’s emotional state is set to be tired. Fig 1 shows an example of the situation, where a posture classified as fatigue is used to improve the user experience.

### User interface adaptation module

In this module, three AUIs are designed. The Automatic AUI suggests user interface adaptations automatically based on the task performed by the user and tries to mitigate user’s frustration with the user interface. The Manual AUI allows the user to adapt a user interface to her/his needs manually. Finally, the Hybrid AUI combines the elements of both Automatic and Manual AUIs. The interaction in all three interfaces includes tasks that involve the layout of the interface, the availability of functionality, and the size of interface elements.

**Fig 5:**
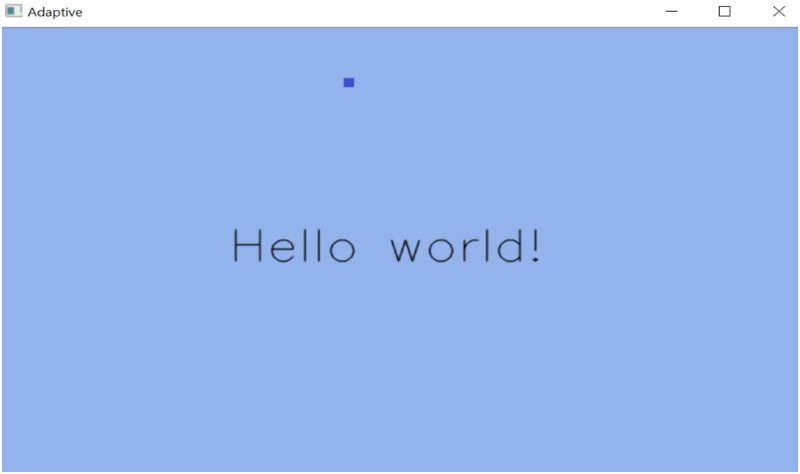
Automatic AUI main window.

**The Automatic AUI:** displays a text in the center as can be seen in Fig 5. The color of the text can be changed using color-buttons. Three color-buttons are available: blue, red, and green, but only the blue button is displayed by default. There is no visible interface element that allows the modification of the user interface.

**Fig 6:**
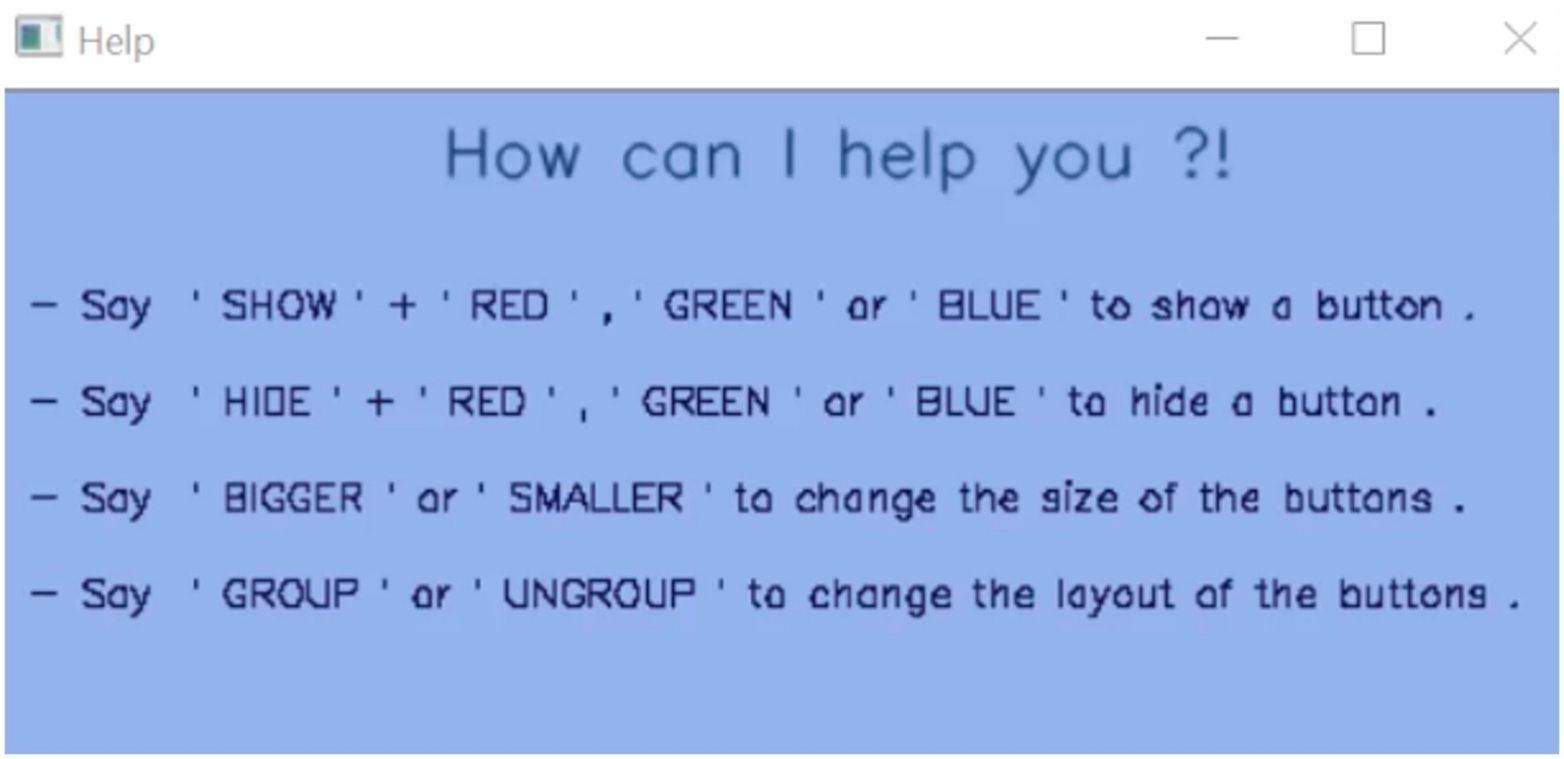
Automatic AUI help window.

The Automatic AUI detects the user’s discomfort as soon as it happens and adapts it appropriately. When the User Emotional State Module detects that the user is feeling a negative emotion, this information is transferred to the Adaption Provider in the UI Adaptation Module. This triggers the display of a help window, as shown in Fig 6, which allows the user to get a better insight about the functionalities that are provided by the interface as well as the adaptation options that are available. The user can then use speech commands to request any adaptation.

**Fig 7:**
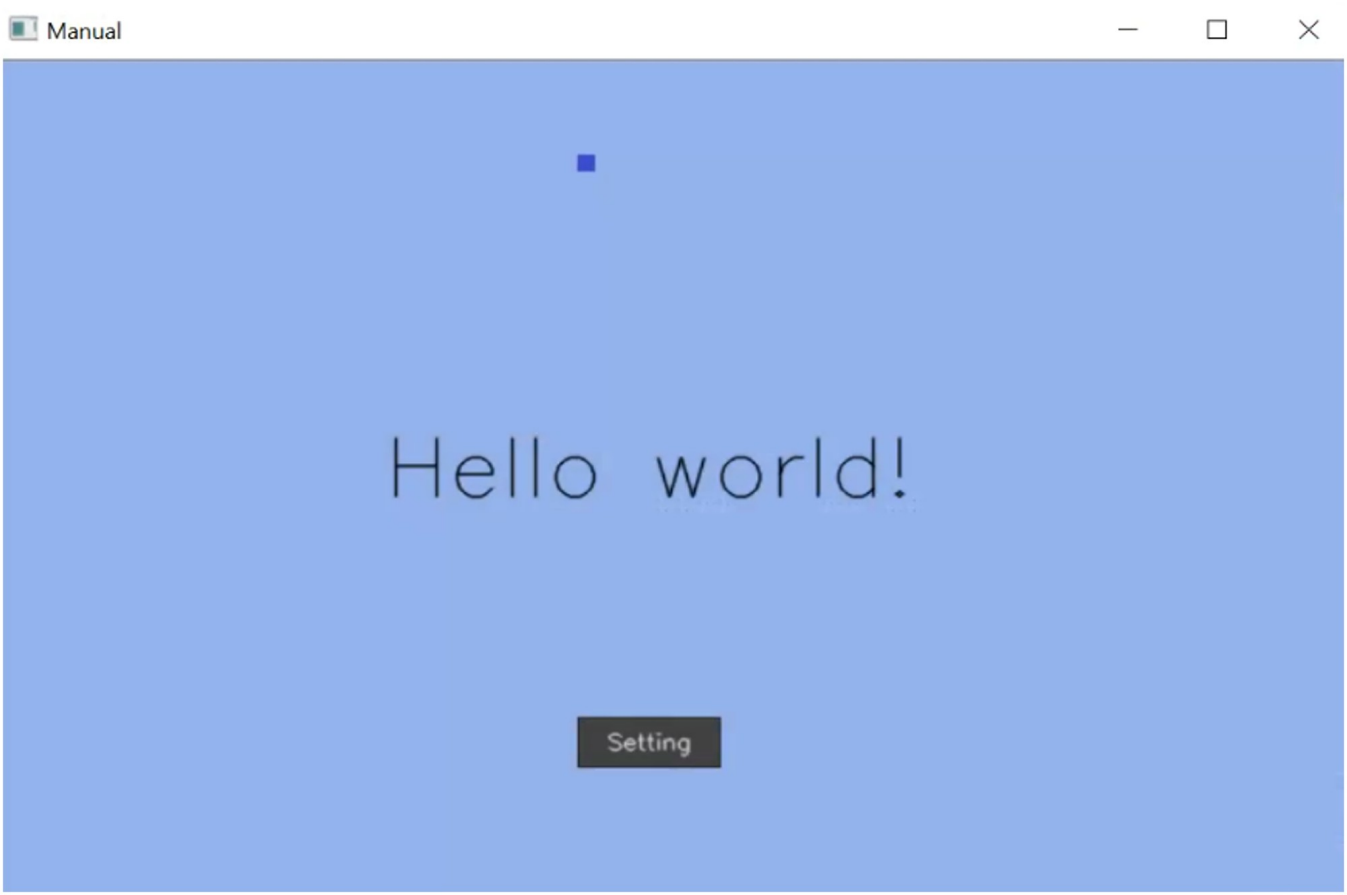
Manual AUI main window.

**Fig 8:**
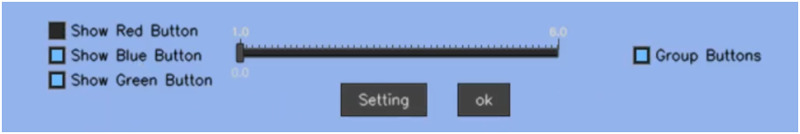
Manual AUI adaptation screen.

**The Manual AUI:** displays the same main screen as the automatic AUI, but the user can adapt the UI by clicking on the Settings button (Fig 7). Theses settings include changing the layout of the interface, change the size of the buttons, or add new functions (new color-buttons), as can be seen in Fig 8.

**The Hybrid AUI:** is a merger between the Automatic and Manual AUIs. Comparing to the Manual AUI, this interface includes the settings-button to allow the user to take desired actions regarding the adaptation. In addition, this interface provides the same adaptation technique as the Automatic AUI depending on the emotion. This interface provides the user with more freedom as to how to apply the adaptation options either by speech commands as in the Automatic AUI or using the mouse as in the Manual AUI.

### Usability and user experience evaluation

To evaluate the performance of the three AUIs, experimental evaluations are performed. Experimental evaluation is one of the techniques that is performed through user participation that generally provides quantitative data. To conduct an experimental evaluation a number of factors have to be defined to ensure a well-structured experiment and reliable results. These factors are detailed below:

1. Participants: 60 participants are selected, 50% female, and 50% male. The participants are divided into three age groups, with age ranging from 18 to 70 years old. More details about the participants are provided in Section ‘Experiment Setup and Testing’.
2. Variables: In the experimental evaluation, two categories of variables are needed to be defined. Independent variables and dependent variables. The independent variables represent the characteristics that can be changed in order to produce different conditions. In this work, the focus is on the layout of the UIs, the size of elements of the UI and provided functionalities. The dependent variables on the other hand, are characteristics that are measured during the experiment. In this work, time taken for each task, and the number of clicks/commands used to complete the task were measured. These measurements will then be used to compute quantitative metrics. More details on these variables are provided in Section ‘Experiment Setup and Testing’.
3. Hypotheses: Considering that the evaluation is conducted to prove a predicted outcome, hypotheses are used to frame this outcome in terms of dependent and independent variables. The first hypothesis states that no difference is observed between conditions. An alternative hypothesis states the exact opposite. Hence, the aim of the experiment is to reject the null hypothesis. In this work, the performance of the AUIs is compared using four metrics: effectiveness, productivity, efficiency, and error safety are computed for each AUI. The details of the metrics can be seen in Section ‘Experiment Setup and Testing’. After the computation, hypotheses were set to evaluate the difference between the metrics for each pair of AUIs. The hypotheses evaluations are detailed in Section ‘Results and Analysis’.
4. Experimental design: An important point that should be considered in an experimental evaluation is to ensure the same conditions for all participants. To minimize the bias or variations, the three group of participants were formed, and each group started the testing for a specific AUI. Details of the experimental design can be seen in Section ‘Experiment Setup and Testing’.

At the end of the experiment, the collected data is represented in terms of mean values for each metric. Repeated-measure ANOVA and one-way ANOVA were employed to analyze this data. Repeated-measure ANOVA is employed to analyze the usability of the three AUIs when evaluated in continuation by the same group of participants. While one-way ANOVA is used to evaluate the data from different viewpoints where each group is independent of the other. More details about the ANOVA analysis is presented in Section ‘Results and Analysis’.

Another evaluation technique that is used in the experiment is the query technique. A questionnaire is used to evaluate user satisfaction regarding the interaction with the three AUIs. The questionnaire provides qualitative data that allows even more insight regarding the experience of the user with each AUI. More details about the questions and the structure of the questionnaire are provided in Section ‘Results and Analysis’. Finally, the session concludes with verbal feedback from the participant about the overall UX and general comments.

As this work requires a user study, it was approved internally first by the Graduate Research Committee at the Department of Computer Science, and then by the College of Graduate Studies at the university level. All participants were 18 years or older and gave their written consent by signing a declaration form that allowed their video recordings and allowed publication of the obtained results along with their images as per the policy outlined in PLOS consent form. They were informed that the recorded data will be saved encrypted, anonymized, and securely managed by authors to be used in scientific publications. The questionnaire used in the study is attached as the support document (S2 Appendix).

## Experiment setup and testing

In this section, all the requirements for the experiment are presented, followed by the workflow. The experiment setup consists of hardware and software components followed by the details of the participants and experimental conditions.

In order to validate the proposed solution, two different experiments were conducted. The first experiment, the accuracy test, allowed the evaluation of the proposed heuristic-based emotion recognition. The second experiment, the usability and UX study, that not only assessed the impact of the adaptation on the UX but also quantified this impact in terms of efficiency, productivity, effectiveness, and error safety. The experiments contain five phases that are detailed in the following sections after the discussion of hardware, software, and participants.

### Hardware and software

**Fig 9:**
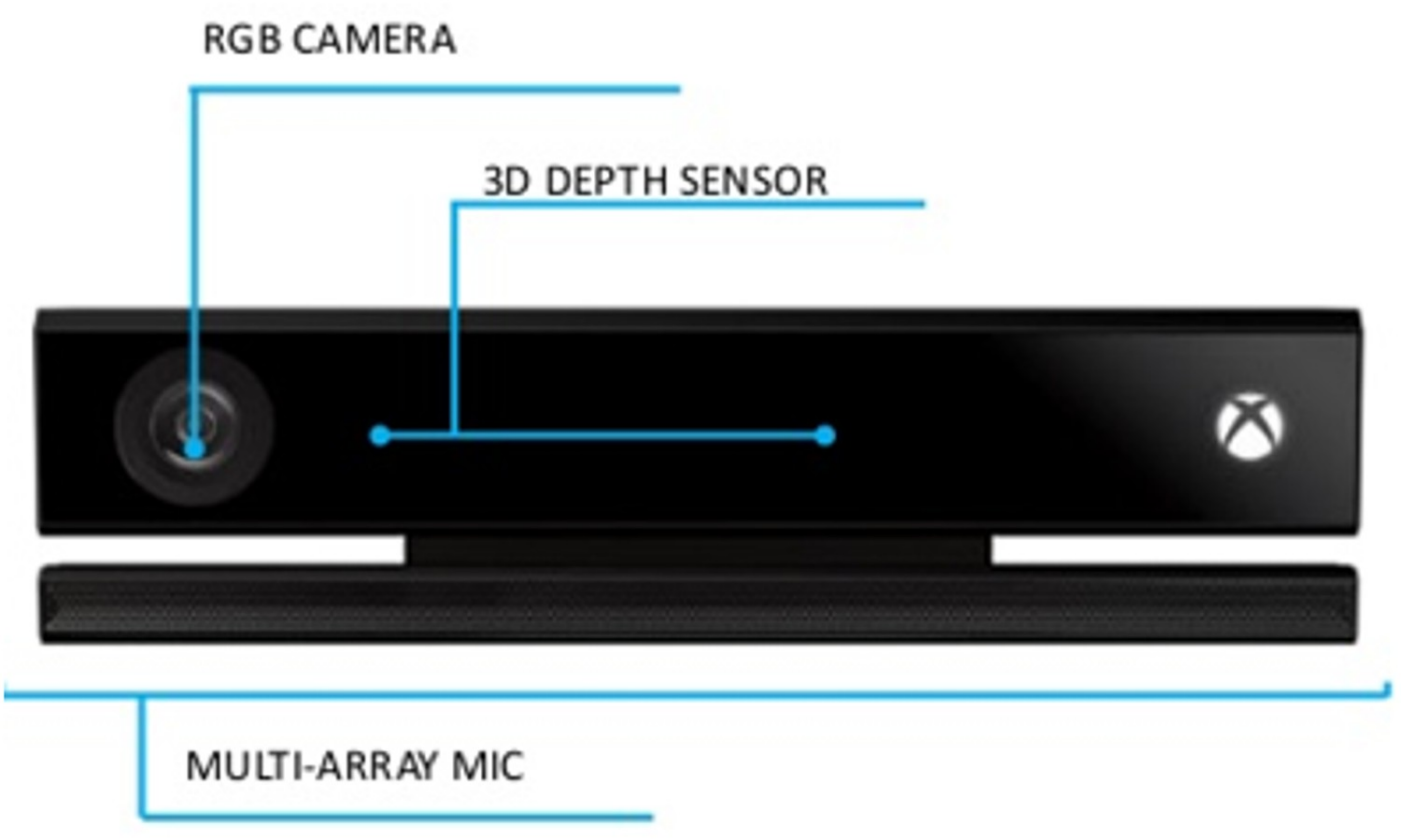
Kinect v2. Depth sensor is in the middle, whereas the microphone array is at the bottom.

This work relies on Kinect v2 (Fig 9) to capture the facial data, posture, and audio for speech recognition of the user. Kinect is equipped with a Full HD (1920x1080) color camera, a low resolution (512x424) depth camera, and an array of four linear microphones. A high-end laptop with Nvidia GeForce 1060 was employed to run the experiments.

The main software tool for acquiring the data is Kinect SDK. The SDK provides drivers, APIs, tools, and code samples in C++ and C#. Along with this SDK, the sensor provides three main streams: a stream of 2D color image frames, a stream of 3D depth image frames, and a stream of 3D skeletal frames.

**Fig 10:**
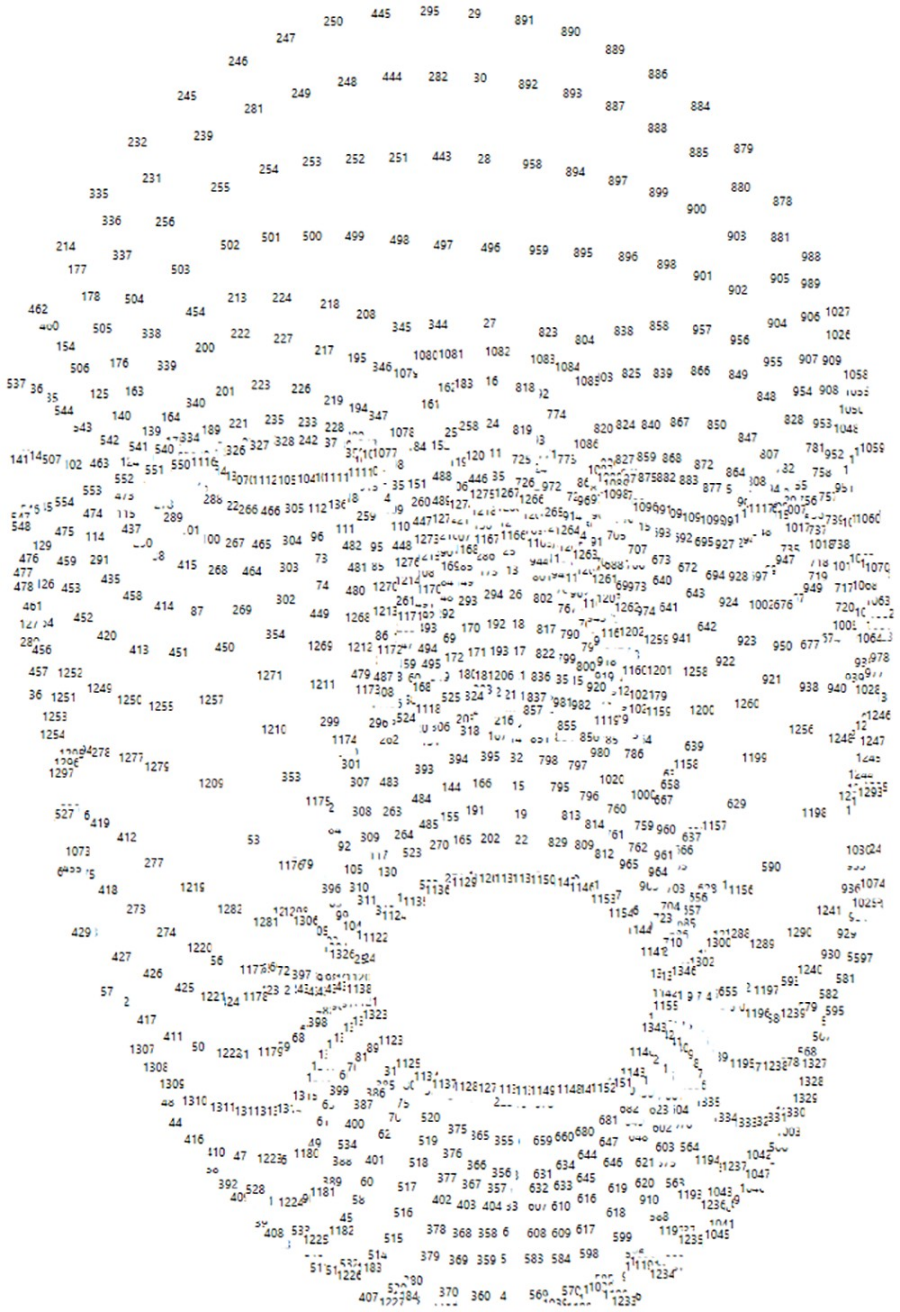
1347 facial point detected by Kinect v2 sensor and Kinect HD Face API.

Kinect HD Face API is one of the APIs provided by the Kinect SDK 2.0. It is considered one of the most advanced face tracking libraries. It allows the capture and real-time tracking of the human face. The face capture API provides a 3D face modal represented as a mesh of 1347 3D vertices (Fig 10). Relying on the face model, it also provides a set of parameters that estimates the shape of the user’s face in terms of Shape Units (SU) referring to the user’s head features such as the mouth, nose, or cheeks. The SUs are weight values varying from -2 to 2, which indicate the variation of the user’s facial attributes from the average face shape. A total of 70 SUs can be retrieved.

The face tracking API provides the face orientation, a head pivot point, and a list of AUs that are based on the same concept as the FACS mentioned in [10], however, both should not be confused since the two are different in terms of facial muscles and the values they record. The face orientation represents the head rotation as a quaternion. The head pivot point defines the center of the user’s head. The AUs represent the computed variation between the neutral face and the user’s face in terms of facial expressions such as mouth is opened, the eyebrow is raised, or eyes are opened. The AUs are numeric weights varying between 0 and 1 except for Jaw Slide Right, Right Eyebrow Lowerer, and Left Eyebrow Lowerer that vary between -1 and +1. A total of 17 AUs are available as discussed in Section ‘Methodology’.

**Fig 11:**
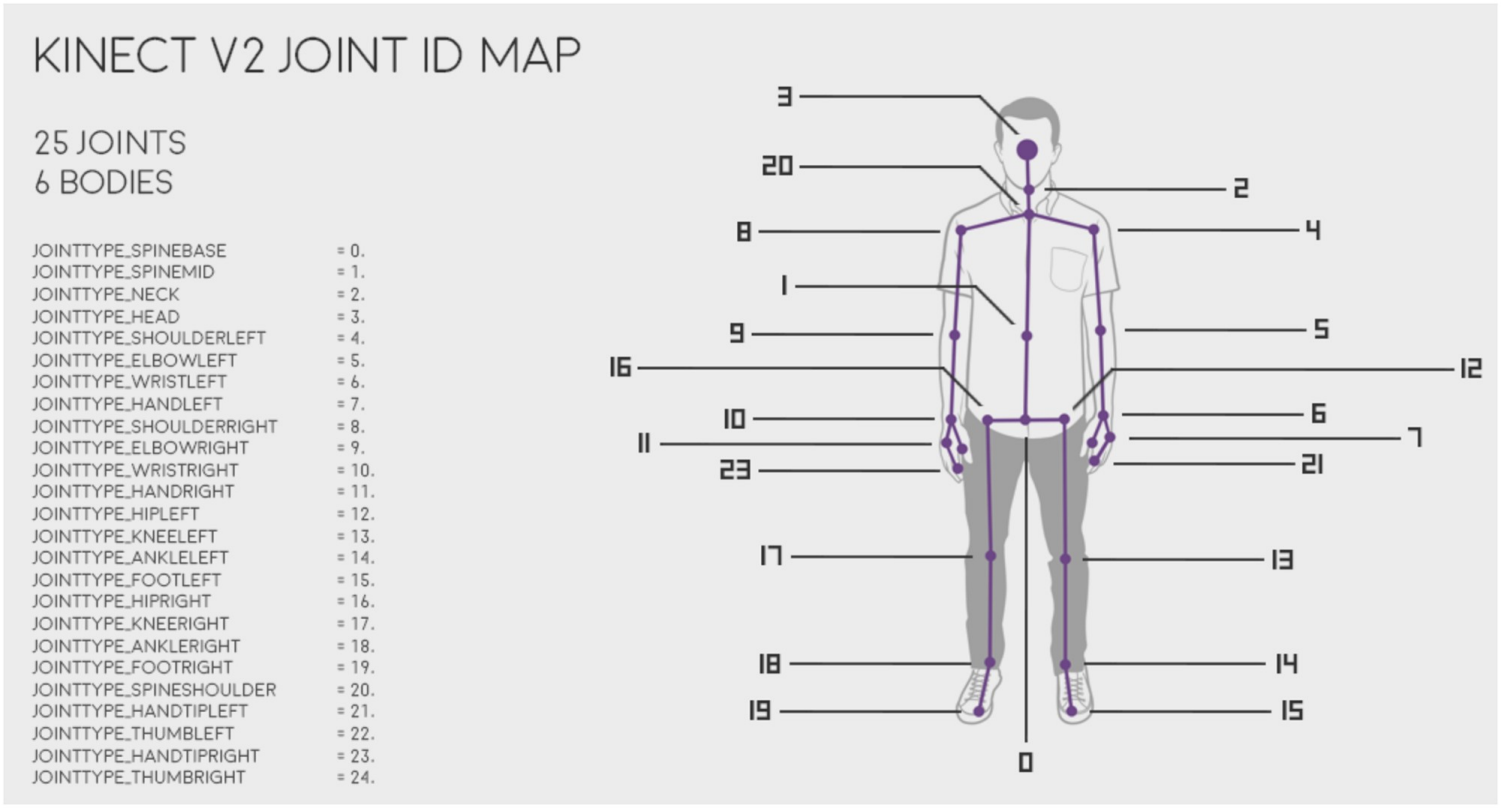
25 joints detected by Kinect v2 sensor and Kinect body API.

Kinect body API allows the detection and tracking of 6 human bodies simultaneously. For each body it provides a set of 25 joints position (Fig 11) and their orientation in three-space. The API also provides information about the state of each hand.

The speech recognition SDK provided in the Kinect SDK 2.0 relies on a speech grammar and a speech recognition engine. The grammar is constructed with a set of rules. These rules define the constraints used by the recognition engine. The speech recognition engine relies on these rules to pair the vocal inputs with the words and phrases within the grammar.

In this work, the AUs obtained from the HD Face API are used as features in the proposed heuristic-based emotion recognition solution for the six basic emotions (Anger, Disgust, Fear, Happiness, Sadness and Surprise). The work also relies on the body tracking API to detect when the user is tired. Also, it uses speech recognition as a voice command input to identify the needs of the user during interaction.

### Participants

For the experiment, 60 volunteers are randomly selected from a larger pool of users agreed to take part in this study. The distribution of the participants is as follows:

1. Total Participants: 60
2. Female: 30
3. Male: 30
4. Age (18–30): 24 (12 Female and 12 Male)
5. Age (31–44): 18 (9 Female and 9 Male)
6. Age (45–70): 18 (9 Female and 9 Male)

**Fig 12:**
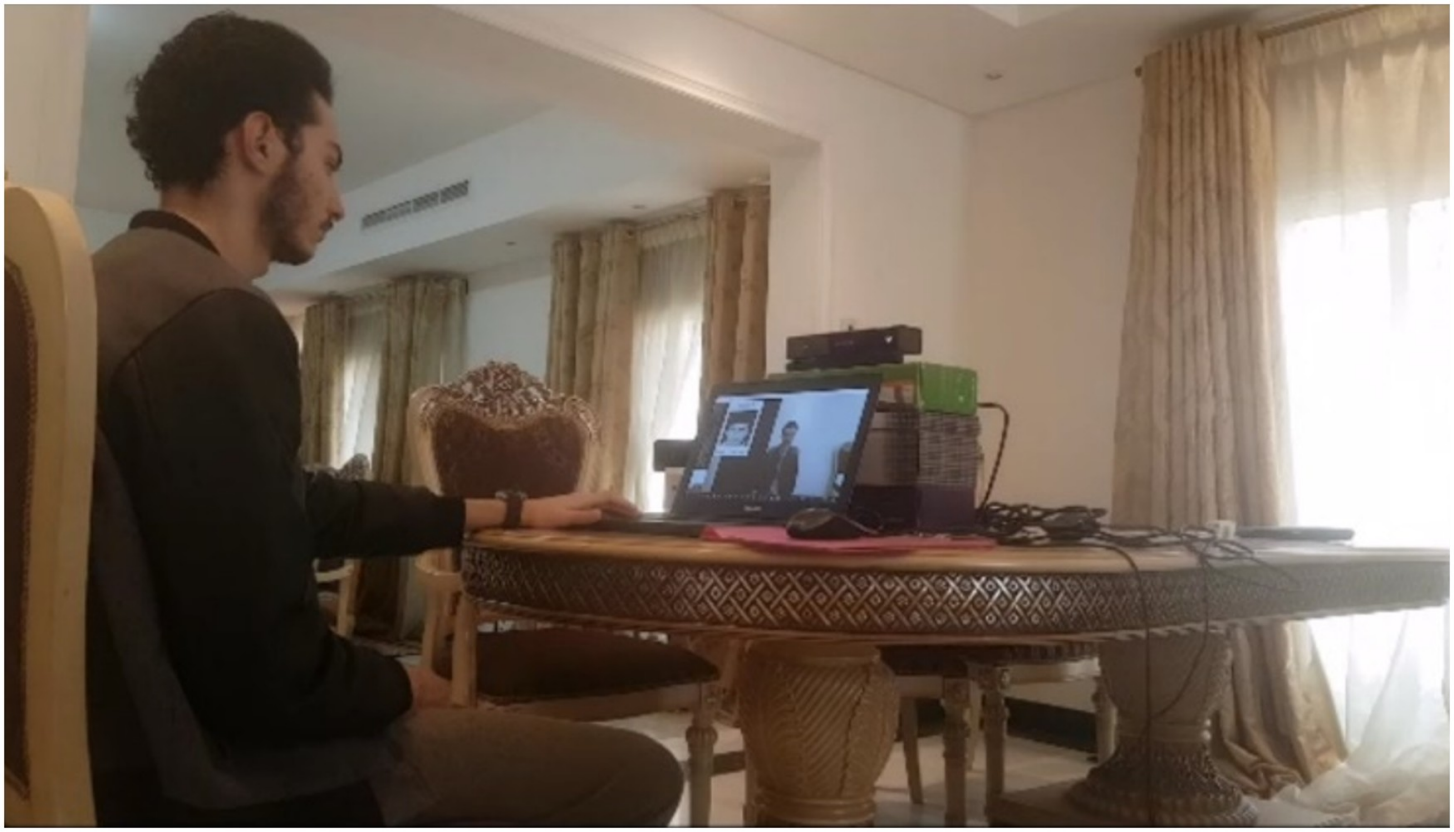
Experiment setup showing one participant.

During the experiment, the participants were seated facing the laptop screen. The Kinect sensor was placed facing the participant at an adequate distance (70 cm). It was ensured that the sensor was properly detecting the face of the participant before the start of the experiment (Fig 12). A significant effort was made into verifying that the frame rate was high enough (> = 26 fps). It was also ensured that the participant was comfortable enough to avoid having any discrepancies during the calibration phase.

### Calibration phase

**Fig 13:**
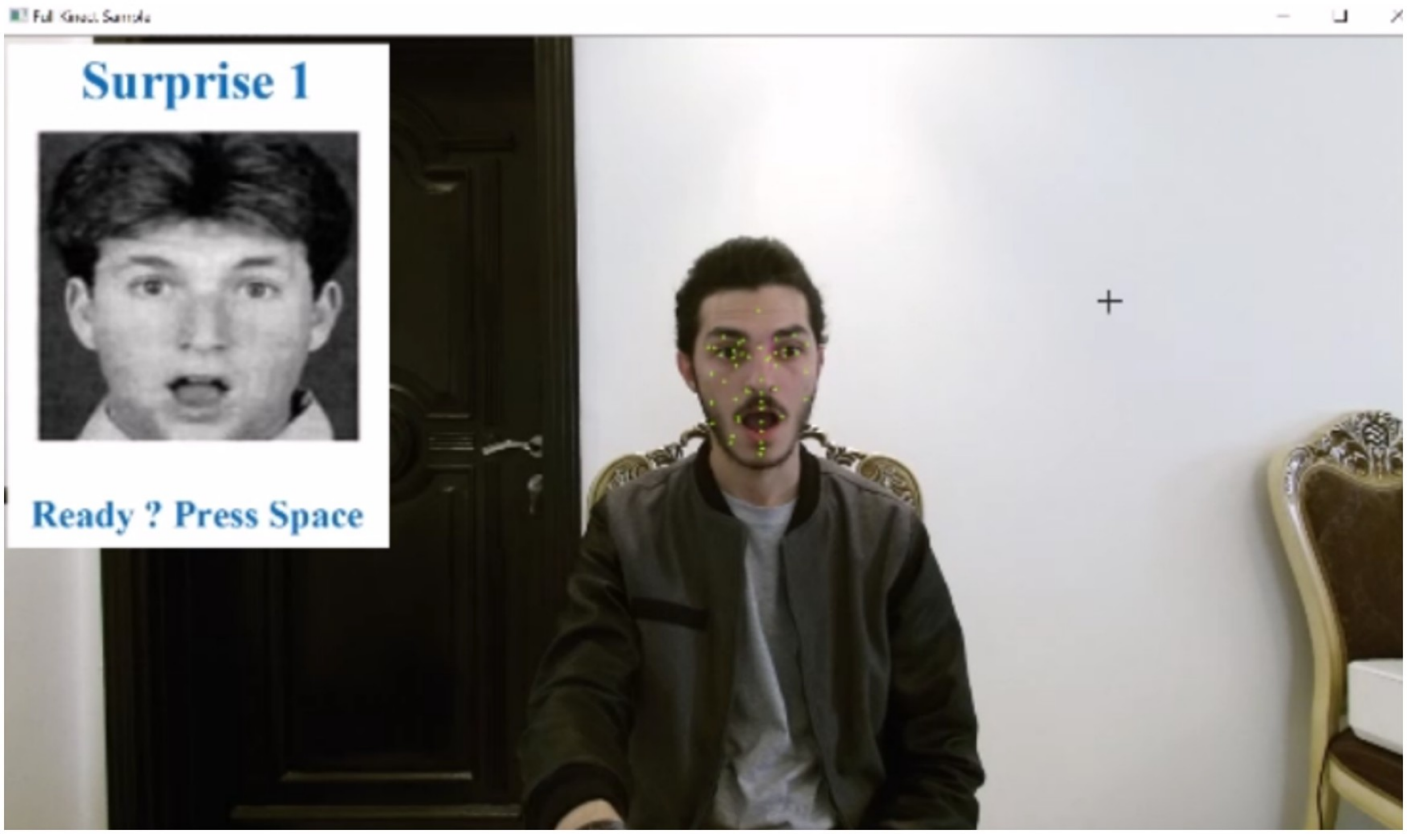
A participant mimicking the surprise emotion during the calibration phase.

In this phase, the system records the facial features of the participants in terms of AUs. For each emotion, the participant is presented with three different pictures that aim at triggering the respective emotion. The user is asked to mimic the displayed emotion. Then the AUs values are recorded for 5 seconds. Fig 13 shows an example of a participant during the calibration phase.

### Emotion recognition accuracy testing

This test is performed after the calibration. In this test, the participant is asked to mimic a specific emotion in their own natural way for 30 seconds. Two confusion matrices are computed to assess the accuracy at which the system was able to recognize each emotion using two classification methods.

One confusion matrix was computed by performing emotion recognition using a direct classification based on the captured AUs at each frame. The second confusion matrix is created after the emotion is recognized using the scoring system explained in the Section ‘Methodology’. The results of the two confusion matrices are detailed in Section ‘Results and Analysis’.

### Usability testing

In this phase, the participant is presented with the three interfaces one at a time. The participants were divided into three groups of 20 participants (10 female and 10 male) each. Following is the composition of each group:

1. Female: 4 (18–24), 3 (31–44), and 3 (45–70)
2. Male: 4 (18–24), 3 (31–44), and 3 (45–70)

Each group evaluated all three AUIs, the only difference being the first AUI is different for each group. This was done to ensure an unbiased first testing of each AUI. Afterward, the participants moved on to the remaining AUIs. Each participant was asked to perform 8 consecutive tasks. The tasks were specifically designed to represent the three independent variables explained in Section ‘Methodology’. Table 2 shows the list of tasks:

**Table 2:** Tasks for evaluating AUIs.

Task	Description
Change Text Color (Blue)	In this task, the blue button is already displayed on the interface space, which makes the action of clicking on the blue button relatively intuitive.
Change Text Color (Red)	In contrast with the previous task, the red button was intentionally hidden from the interface space. This has been done to trigger the frustration of the user.
Display Button (Green)	In this task the user is required to adapt the UI by adding a new functionality to the UI.
Hide Button (Red)	In this task the user is required to adapt the UI by hiding a functionality.
Group Buttons	In this task the user is required to adapt the UI by changing the layout of the UI.
Increase Button Size	In this task the user is required to adapt the UI by enhancing UI elements.
Decrease Button Size	This task has the same intentions as the previous task. By increasing and decreasing the size of the buttons, the user is able to adapt the UI elements to the size the fits the needs of the user. This task has the same intentions as the previous task. By increasing and decreasing the size of the buttons, the user is able to adapt the UI elements to the size the fits the needs of the user.
Ungroup Buttons	This task has the same intentions as the grouping task. By grouping and ungrouping the buttons, the user is able to adapt the layout of the UI to fit the needs of the user.

For each task, the time to complete each task as well as the number of correct and incorrect clicks/commands were simultaneously recorded. The recorded data was then used to compute different metrics that allowed the assessment of the usability of each interface. The formulas to calculate the different metrics were acquired from [40]. The metrics and the parameters necessary to calculate them are detailed below:

**Effectiveness:** accuracy and completeness with which users achieve specified goals. In other words, the effectiveness allows us to evaluate if the interface performs the tasks it is intended to do. It is calculated as follow:

$$\frac{Min\#of\;correct\;actions\;required\;to\;complete\;the\;task}{\#of\;correct\;actions\;performed+\#of\;incorrect\;actions\;performed}$$

**Productivity:** the ratio between the functional values of software produced to the labor and expense of producing it. It allows to evaluate if the interface performs the tasks it is intended to do in an optimal time. It is calculated as follows:

$$\frac{Min\#of\;correct\;actions\;required\;to\;complete\;the\;task}{Duration\;period}$$

**Efficiency:** resources expended in relation to the accuracy and completeness with which users achieve goals. The efficiency allows us to evaluate if the interface performs as it is intended and properly within a certain duration. It is calculated as follows:

$$\frac{Effectiveness}{Duration\;period}$$

**Error Safety:** the degree to which a system protects users against making errors. The error safety allows us to evaluate if the user can recover from errors easily. It is calculated as follows:

$$\frac{\#of\;incorrect\;actions\;performed}{\#of\;correct\;actions\;performed+\#of\;incorrect\;actions\;performed}$$

### Questionnaire

At the end of the experiment, the participants were asked to fill a comprehensive questionnaire. This questionnaire allowed us to get complementary information about the experience that the participant had with each interface. The questionnaire contains three main categories of questions:

1. General Questions
2. Task-specific Questions
3. Comparative Questions

The questionnaire is followed by a semi-structured interview. The questionnaire details and the responses are discussed in Section ‘Results and Analysis’.

### Scenarios

To further clarify the proposed experiment workflow and how the components of the proposed solution intertwine during an experiment, three scenarios are described in detail. These scenarios detail the first two tasks that the user performs for each AUI.

**Automatic AUI:** First the participant is asked to change the text color to blue. The blue button is already on the UI space which makes the task relatively intuitive. Then the participant is asked to change the color to red, but this time the red button is not displayed on the UI space, which quickly becomes a source of frustration to the participant considering that this AUI is a completely automatic system that only adapts through the Help Provider. As soon as the participant shows a negative emotion, the help window is displayed, and the participant identifies and executes the desired adaptation through a speech command.

**Manual AUI:** Similarly, in the Manual AUI the participant is asked to change the text color to blue then to red, but since the red button is not available in the UI space, the participant has to click on the setting button to display it. However, in some cases the participant starts to experience a negative emotion about the unavailability of the functionality but the Manual AUI is not equipped with an automatic adaptation, so nothing is done to provide help.

**Hybrid AUI:** Automatic and Manual AUIs are combined to form the Hybrid AUI. In this AUI, either the user can intuitively click on the setting button, or the automatic help is quickly provided when the user shows a negative emotion.

At the end of the experiment, the participants were asked to fill a comprehensive questionnaire (S2 Appendix) followed by the semi-structured interview.

## Results and analysis

As explained in the previous section, all three AUIs were tested in two stages. In the first stage, the accuracy of the emotion recognition was established. First, emotion recognition accuracy results are presented. Afterward, the completion time and error rate are used to derive the usability score for each AUI are detailed. The hypothesis evaluation for the viability of each AUI in terms of effectiveness, productivity, efficiency, and error safety is presented after the completion time. The same data is used to analyze the results from different viewpoints of both genders and the three age groups. The usability and user experience for the AUIs was also validated using a questionnaire. The results and analysis of the questionnaire are presented at the end of this section.

### Emotion recognition accuracy results

**Fig 14:**
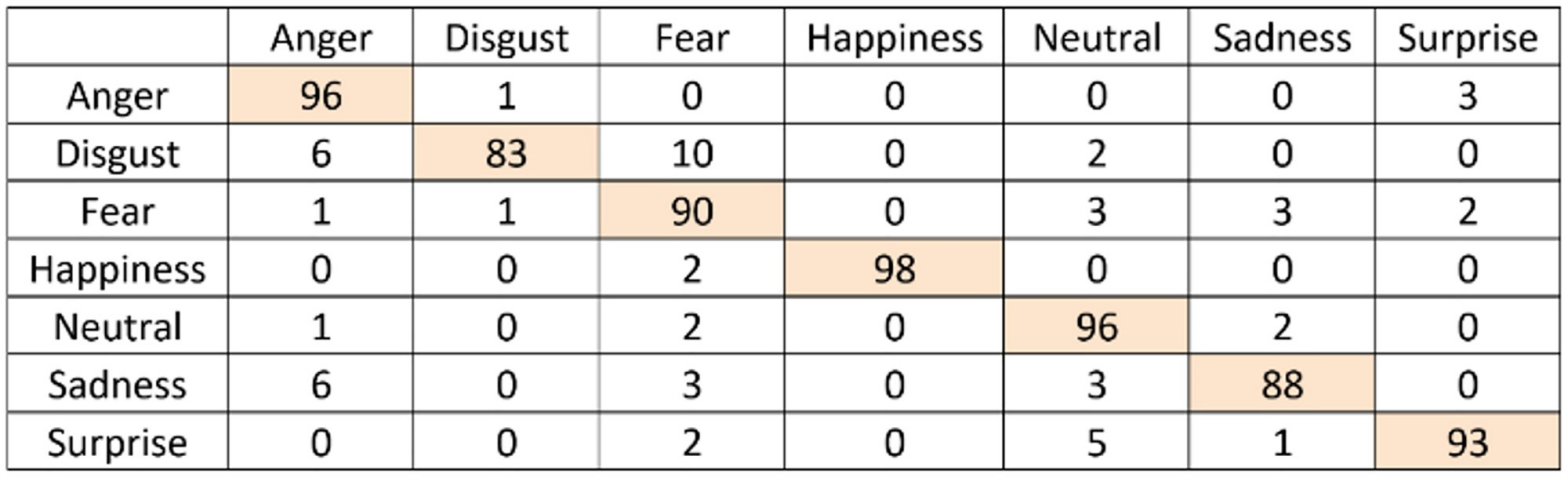
Confusion matrix for emotion recognition with the scoring system.

**Fig 15:**
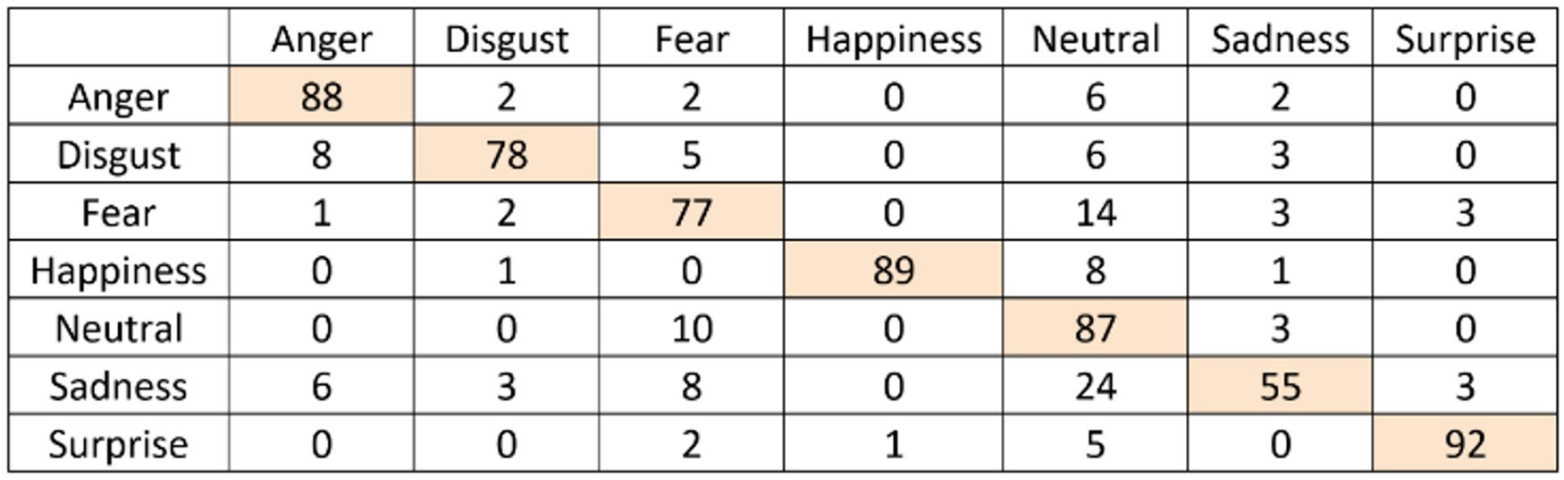
Confusion matrix for emotion recognition without the scoring system.

The confusion matrix for the emotion recognition with or without the scoring system can be seen in Figs 14 and 15. As explained in the methodology section, the scoring system counts the most observed emotion with a time window and assigns the emotion based on the maximum number of counts. This is done to minimize the tracking failures that result from the failures of the underlying algorithm in Kinect Face Tracking SDK that was employed to capture the face of the person. As an additional challenge, getting 6 different emotions from Kinect is a challenging task because some of the emotions are very close to each other in terms of facial expression.

As can be seen in Fig 14, the scoring system works extremely well in recognizing the emotions to a very high degree of accuracy, whereas the accuracy of the emotion recognition drops if the scoring system is not employed. Employing the proposed scoring system resulted in very accurate emotion recognition. During the tests, no issues were found for the Adaptive and Hybrid AUIs. Users did not experience any confusion that would have been caused by an incorrect recognition.

### Completion time

**Fig 16:**
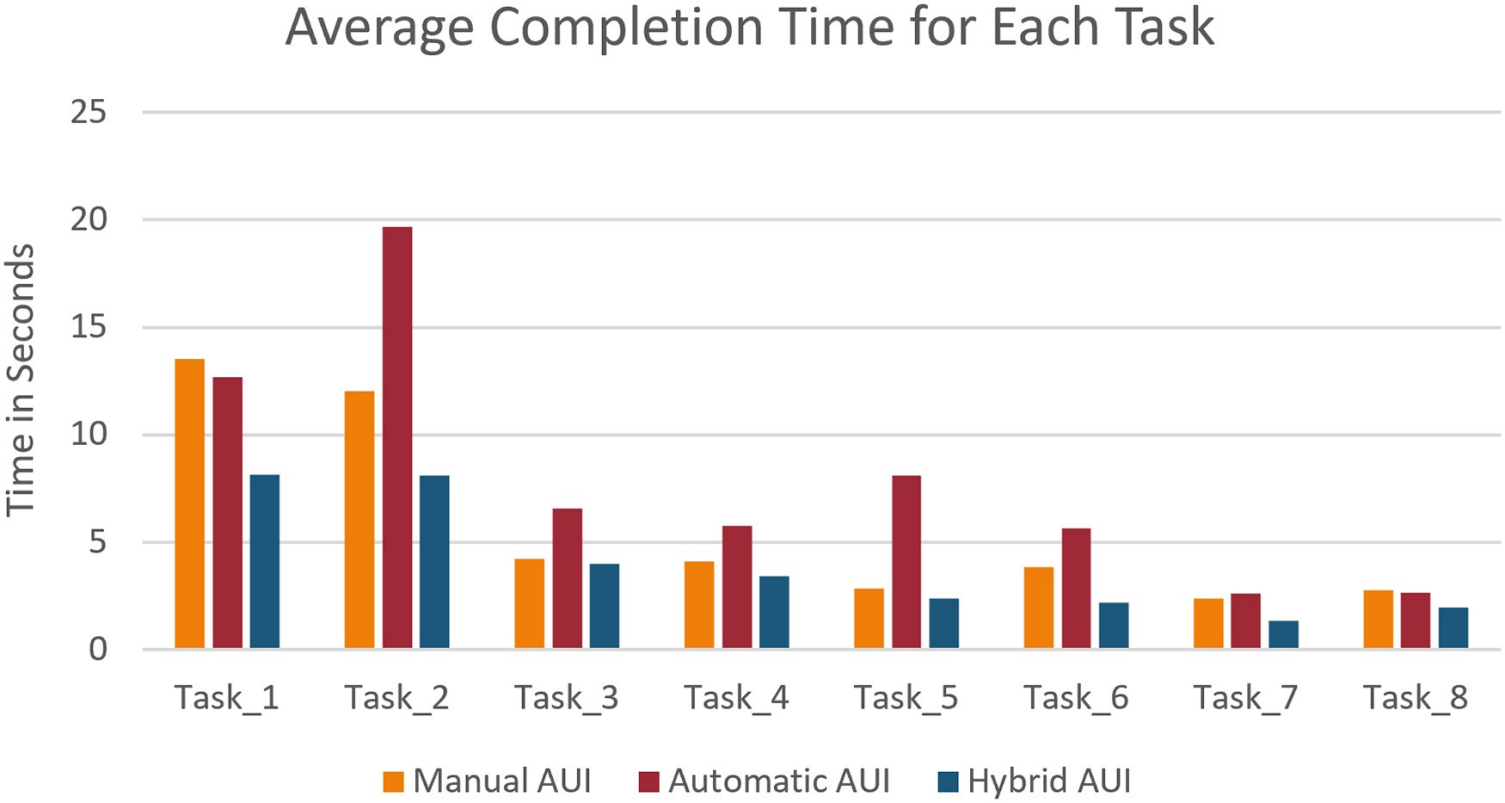
Average completion time for each AUI and each task.

**Fig 17:**
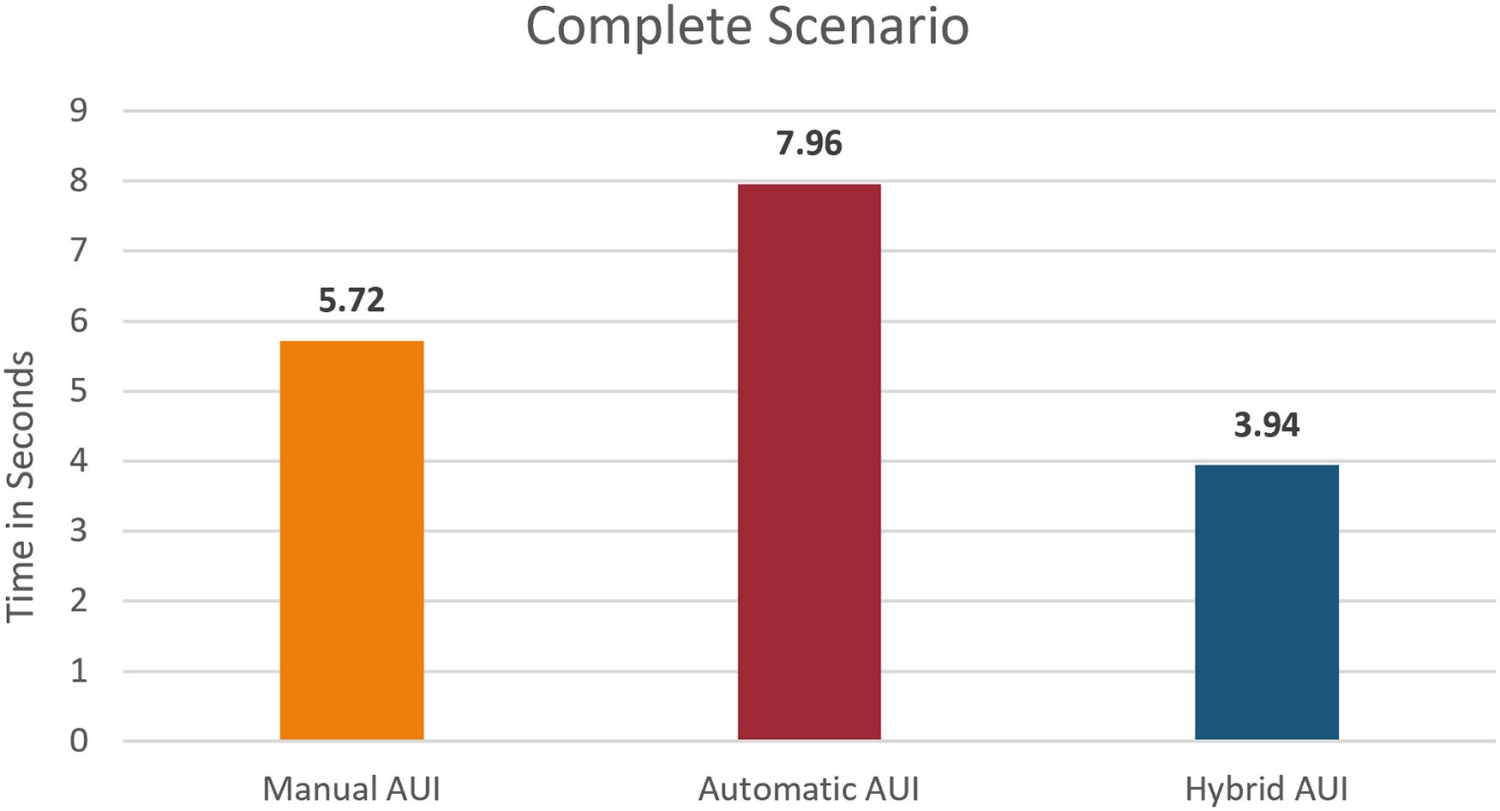
Average completion time for each AUI and all tasks.

The average completion time for each task can be seen in Fig 16. As explained in the Table 2 in the ‘Usability Testing’ section. The second task is meant to trigger the level of frustration of the user during the execution of this task. This is reflected through the completion time of the task that is the highest times, especially for the Automatic AUI. The three interfaces were also compared in terms of time taken by the participants to complete all 8 tasks, as can be seen Fig 17.

In general, as it can be observed that using the Automatic AUI takes most of the time. This is because it is a completely automatic system that relies on the correct detection of the emotions of the user. As the user does not find any way to modify the system, it takes some time for the emotion to trigger and detected before the adaptation can take place. On the other hand, as will be seen in the hypothesis evaluation, and the UX survey that extra time does not necessarily induce a poor UX for the Manual and Hybrid AUIs. The familiarity of the user with the existing UI and the feeling of being in control plays an important part.

### Hypothesis evaluation

As explained in the previous sections, the hypothesis testing relies on refuting or supporting a proposed hypothesis. In general, the hypothesis is set to promote one interface compared to another. First, a null hypothesis is defined which needs to be refuted to have the alternative hypothesis automatically supported.

In this work we compare three AUIs in terms of their effectiveness, productivity, efficiency, and error safety. As explained in the ‘Usability’ section that we divide the 60 participants into three groups of 20, and each group has an equal number of representations from both genders and all three age groups. Each group tested all three AUIs, but the starting AUI was different to ensure an unbiased evaluation of each AUI without a prior learning experience. This resulted in three testing scenarios comprising of a unique combination of three AUIs that were evaluated one after the other by three independent groups of 20 participants. Each AUI was evaluated with eight tasks that were detailed in Table 2 in the ‘Usability Testing’ section.

For each task, the number of actions, correct and incorrect, and time taken to complete the task were recorded. As described in the ‘Usability Testing’ section, the usability parameters: effectiveness and error safety are calculated from the number of correct and incorrect actions, whereas the productivity and efficiency also depend on the task completion time. After analyzing the data from the 60 participants, we found out that the difference in the number of correct and incorrect actions between the participants was minimal and all three AUIs were very similar in terms of their effectiveness and error safety. The data analysis did not identify any statistical differences between these two measures. On the other hand, as shown in the previous section, there is a significant difference in the task completion times, and overall completion times for each AUI. As shown later in this section, repeated-measure ANOVA and one-way ANOVA identified significant differences between AUIs in terms of their productivity and efficiency.

Thus, we define the null hypothesis for productivity and efficiency as follows: There is no statistically significant difference between the means of each AUI in terms of their task completion time. We also define the alternative hypothesis which is to be supported if the null hypothesis is refuted as follows: There is a statistically significant difference between the means of each AUI in terms of their task completion time. Similar null and alternative hypotheses are adopted later to compare the data from both genders and different age groups. Using repeated-measures ANOVA or one-way ANOVA we identify if the null hypothesis can be refuted or not. Please note that we used alpha = 0.05 for ANOVA. If the null hypothesis is refuted, then we perform Tukey’s HSD test to identify the specific AUIs that are significantly different. In the following sections, we will present all the findings from these analyses.

### Repeated-measures ANOVA

We have three groups of 20 participants, where each group evaluated three AUIs the only difference being the different starting AUI:

1. Group #1: Started with Manual AUI
2. Group #2: Started with Automatic AUI
3. Group #3: Started with Hybrid AUI

For each group, we conduct repeated-measures ANOVA on the task completion time to identify if there is a statistically significant difference between the means of three AUIs.

**Group #1:** Table 3 shows a summary of the group’s data for each AUI. Table 4 shows the results from repeated-measures ANOVA for this group.

**Table 3:** Data summary—Group #1.

Treatment	Subject Count	Mean	Variance
Manual	20	5.80	0.70
Automatic	20	8.03	0.41
Hybrid	20	3.76	0.31

**Table 4:** Repeated-measures ANOVA—Group #1.

*Source of Variation*	*SS*	*df*	*MS*	*F*	*P-value*	*F crit*
Treatments (AUIs)	181.82	2	90.91	906.01	8.70E-33	3.24
Subjects	23.20	19	1.22	12.17	8.11E-11	1.86
Error	3.81	38	0.10			
Total	208.83	59				

As can be seen in Table 4, there is a significant effect of AUIs on participants’ task completion time, F(2, 38) = 3.24, p < 0.05. Therefore, the null hypothesis is refuted, and the alternative hypothesis is supported. Using Tukey’s HSD test the value of HSD is calculated: HSD = 0.24. As the absolute difference between the means of each AUI is more than the HSD, it shows that each AUI is statistically different compared to the other in terms of the task completion time. It shows that for Group #1, the Hybrid AUI is most productive and efficient.

**Group #2:** Table 5 shows a summary of the group’s data for each AUI. Table 6 shows the results from repeated-measures ANOVA for this group.

**Table 5:** Data summary—Group #2.

Treatment	Subject Count	Mean	Variance
Automatic	20	8.28	1.23
Manual	20	6.1	0.50
Hybrid	20	3.64	0.83

**Table 6:** Repeated-measures ANOVA—Group #2.

*Source of Variation*	*SS*	*df*	*MS*	*F*	*P-value*	*F crit*
Treatments (AUIs)	215.31	2	107.65	982.70	1.91E-33	3.24
Subjects	14.39	19	0.75	6.91	2.41E-07	1.86
Error	4.16	38	0.10			
Total	233.87	59				

As can be seen in Table 6, there is a significant effect of AUIs on participants’ task completion time, F(2, 38) = 3.24, p < 0.05. Therefore, the null hypothesis is refuted, and the alternative hypothesis is supported. Using Tukey’s HSD test the value of HSD is calculated: HSD = 0.25. As the absolute difference between the means of each AUI is more than the HSD, it shows that each AUI is statistically different compared to the other in terms of the task completion time. It shows that for Group #2, the Hybrid AUI is most productive and efficient.

**Group #3:** Table 7 shows a summary of the group’s data for each AUI. Table 8 shows the results from repeated-measures ANOVA for this group.

**Table 7:** Data summary—Group #3.

Treatment	Subject Count	Mean	Variance
Hybrid	20	4.43	1.14
Automatic	20	7.57	2.21
Manual	20	5.26	1.12

**Table 8:** Repeated-measures ANOVA—Group #3.

*Source of Variation*	*SS*	*df*	*MS*	*F*	*P-value*	*F crit*
Treatments (AUIs)	106.02	2	53.01	181.26	3.67E-20	3.24
Subjects	17.11	19	0.90	3.07	1.00E-3	1.86
Error	11.11	38	0.29			
Total	134.24	59				

As can be seen in Table 8, there is a significant effect of AUIs on participants’ task completion time, F(2, 38) = 3.24, p < 0.05. Therefore, the null hypothesis is refuted, and the alternative hypothesis is supported. Using Tukey’s HSD test the value of HSD is calculated: HSD = 0.41. As the absolute difference between the means of each AUI is more than the HSD, it shows that each AUI is statistically different compared to the other in terms of the task completion time. It shows that for Group #3, the Hybrid AUI is most productive and efficient.

**Summary:** In summary, after analyzing the results from all three groups, it can be stated that the Hybrid AUI achieves better performance than both Manual and Automatic AUI in terms of productivity and efficiency, whereas all AUIs performed similarly in terms of effectiveness and error safety. Manual AUI though less productive and efficient than the Hybrid AUI, still is more productive and efficient compared to the Automatic AUI.

### Gender and age group analysis using one-way ANOVA

We also evaluated the data from the viewpoints of both genders, comparatively and individually, and also for each age group, comparatively and individually. Unlike the groups identified in the previous section where three AUIs were evaluated one after the other, the gender or age group data is completely independent. Therefore, we analyzed this data using one-way ANOVA. Similar null and alternative hypotheses are employed as explained in the previous section, followed by Tukey’s HSD test if the null hypothesis is rejected.

**Viewpoint #1: Subjects = Female and Treatments = AUI:** Table 9 shows the summary of this viewpoint. Table 10 shows the results from one-way ANOVA for this viewpoint.

**Table 9:** Data summary—Viewpoint #1.

Treatment	Subject Count	Mean	Variance
Manual	30	5.80	0.80
Automatic	30	7.87	1.64
Hybrid	30	3.94	0.24

**Table 10:** One-way ANOVA—Viewpoint #1.

*Source of Variation*	*SS*	*df*	*MS*	*F*	*P-value*	*F crit*
Treatments (AUIs)	232.30	2	116.15	251.18	7.19E-37	3.10
Subjects	40.22	87	0.46			
Total	272.53	89				

As can be seen in Table 10, there is a significant effect of AUIs on female participants’ task completion time, F(2, 87) = 3.10, p < 0.05. Therefore, the null hypothesis is refuted, and the alternative hypothesis is supported. Using Tukey’s HSD test the value of HSD is calculated: HSD = 0.42. As the absolute difference between the means of each AUI is more than the HSD, it shows that each AUI is statistically different compared to the other in terms of the task completion time. It shows that for Viewpoint #1, the Hybrid AUI is most productive and efficient.

**Viewpoint #2: Subjects = Male and Treatments = AUI:** Table 11 shows a summary of this viewpoint. Table 12 shows the results from one-way ANOVA for this viewpoint.

**Table 11:** Data summary—Viewpoint #2.

Treatment	Subject Count	Mean	Variance
Manual	30	5.64	1.25
Automatic	30	8.05	1.38
Hybrid	30	3.95	0.45

**Table 12:** One-way ANOVA—Viewpoint #2.

*Source of Variation*	*SS*	*df*	*MS*	*F*	*P-value*	*F crit*
Treatments (AUIs)	254.17	2	127.08	212.10	3.51E-34	3.10
Subjects	52.13	87	0.59			
Total	306.30	89				

As can be seen in Table 12, there is a significant effect of AUIs on male participants’ task completion time, F(2, 87) = 3.10, p < 0.05. Therefore, the null hypothesis is refuted, and the alternative hypothesis is supported. Using Tukey’s HSD test the value of HSD is calculated: HSD = 0.47. As the absolute difference between the means of each AUI is more than the HSD, it shows that each AUI is statistically different compared to the other in terms of the task completion time. It shows that for Viewpoint #2, the Hybrid AUI is most productive and efficient.

**Viewpoint #3: Subjects = Manual AUI Task Completion Time and Treatments = Genders:** Table 13 shows a summary of this viewpoint. Table 14 shows the results from one-way ANOVA for this viewpoint.

**Table 13:** Data summary—Viewpoint #3.

Treatment	Subject Count	Mean	Variance
Female	30	5.80	0.80
Male	30	5.64	1.25

**Table 14:** One-way ANOVA—Viewpoint #3.

*Source of Variation*	*SS*	*df*	*MS*	*F*	*P-value*	*F crit*
Treatments (Genders)	0.35	1	0.35	0.40	0.52	4.00
Subjects	51.08	58	0.88			
Total	51.44	59				

As can be seen in Table 14, there is no significant effect of genders on participants’ Manual AUI task completion time, F(1, 58) = 4.00, p < 0.05. Therefore, we cannot refute the null hypothesis. It shows that for Viewpoint #3, both genders perform with a similar level of productivity and efficiency.

**Viewpoint #4: Subjects = Automatic AUI Task Completion Time and Treatments = Genders:** Table 15 shows a summary of this viewpoint. Table 16 shows the results from one-way ANOVA for this viewpoint.

**Table 15:** Data summary—Viewpoint #4.

Treatment	Subject Count	Mean	Variance
Female	30	7.87	1.64
Male	30	8.05	1.38

**Table 16:** One-way ANOVA—Viewpoint #4.

*Source of Variation*	*SS*	*df*	*MS*	*F*	*P-value*	*F crit*
Treatments (Genders)	0.46	1	0.46	1.26	0.26	4.00
Subjects	21.01	58	0.36			
Total	21.47	59				

As can be seen in Table 16, there is no significant effect of genders on participants’ Automatic AUI task completion time, F(1, 58) = 4.00, p < 0.05. Therefore, we cannot refute the null hypothesis. It shows that for Viewpoint #4, both genders perform with a similar level of productivity and efficiency.

**Viewpoint #5: Subjects = Hybrid AUI Task Completion Time and Treatments = Genders:** Table 17 shows a summary of this viewpoint. Table 18 shows the results from one-way ANOVA for this viewpoint.

**Table 17:** Data summary—Viewpoint #5.

Treatment	Subject Count	Mean	Variance
Female	30	3.94	0.24
Male	30	3.95	0.45

**Table 18:** One-way ANOVA—Viewpoint #5.

*Source of Variation*	*SS*	*df*	*MS*	*F*	*P-value*	*F crit*
Treatments (Genders)	2.00E-3	1	2.00E-3	6.00E-3	0.93	4.00
Subjects	20.25	58	0.35			
Total	20.26	59				

As can be seen in Table 18, there is no significant effect of genders on participants Hybrid AUI task completion time, F(1, 58) = 4.00, p < 0.05. Therefore, we cannot refute the null hypothesis. It shows that for Viewpoint #5, both genders perform with a similar level of productivity and efficiency.

**Viewpoint #6: Subjects = Age Group 18–30 and Treatments = AUI:** Table 19 shows a summary of this viewpoint. Table 20 shows the results from one-way ANOVA for this viewpoint.

**Table 19:** Data summary—Viewpoint #6.

Treatment	Subject Count	Mean	Variance
Manual	24	4.32	0.59
Automatic	24	7.4	1.33
Hybrid	24	3.93	0.41

**Table 20:** One-way ANOVA—Viewpoint #6.

*Source of Variation*	*SS*	*df*	*MS*	*F*	*P-value*	*F crit*
Treatments (AUIs)	173.53	2	86.76	268.16	2.89E-33	3.12
Subjects	22.32	69	0.32			
Total	195.86\	71				

As can be seen in Table 20, there is a significant effect of AUIs on participants in the age range of 18–30 task completion time, F(2, 69) = 3.12, p < 0.05. Therefore, the null hypothesis is refuted, and the alternative hypothesis is supported. Using Tukey’s HSD test the value of HSD is calculated: HSD = 0.40. As the absolute difference between the means of Manual and Hybrid AUI is less than HSD, it shows that statistically, the Manual and Hybrid AUI are similar for this age group. On the other hand, the Automatic AUI is statistically the least productive and efficient for this age group.

**Viewpoint #7: Subjects = Age Group 31–44 and Treatments = AUI:** Table 21 shows a summary of this viewpoint. Table 22 shows the results from one-way ANOVA for this viewpoint.

**Table 21:** Data summary—Viewpoint #7.

Treatment	Subject Count	Mean	Variance
Manual	18	5.94	0.54
Automatic	18	8.1	1.26
Hybrid	18	3.95	0.28

**Table 22:** One-way ANOVA—Viewpoint #7.

*Source of Variation*	*SS*	*df*	*MS*	*F*	*P-value*	*F crit*
Treatments (AUIs)	155.50	2	77.75	144.68	9.51E-22	3.17
Subjects	27.40	51	0.53			
Total	182.91	53				

As can be seen in Table 22, there is a significant effect of AUIs on participants’ in the age range of 31–44 task completion time, F(2, 51) = 3.17, p < 0.05. Therefore, the null hypothesis is refuted, and the alternative hypothesis is supported. Using Tukey’s HSD test the value of HSD is calculated: HSD = 0.58. As the absolute difference between the means of each AUI is more than the HSD, it shows that each AUI is statistically different compared to the other in terms of the task completion time. As a result, for Viewpoint #7, the Hybrid AUI is most productive and efficient.

**Viewpoint #8: Subjects = Age Group 45–70 and Treatments = AUI:** Table 23 shows a summary of this viewpoint. Table 24 shows the results from one-way ANOVA for this viewpoint.

**Table 23:** Data summary—Viewpoint #8.

Treatment	Subject Count	Mean	Variance
Manual	18	6.9	0.59
Automatic	18	8.26	2.20
Hybrid	18	3.96	0.16

**Table 24:** One-way ANOVA—Viewpoint #8.

*Source of Variation*	*SS*	*df*	*MS*	*F*	*P-value*	*F crit*
Treatments (AUIs)	173.05	2	86.52	311.23	2.63E-29	3.17
Subjects	14.17	51	0.27			
Total	187.23	53				

As can be seen in Table 24, there is a significant effect of AUIs on participants in the age range of 45–70 task completion time, F(2, 51) = 3.17, p < 0.05. Therefore, the null hypothesis is refuted, and the alternative hypothesis is supported. Using Tukey’s HSD test the value of HSD is calculated: HSD = 0.42. As the absolute difference between the means of each AUI is more than the HSD, it shows that each AUI is statistically different compared to the other in terms of the task completion time. Hence, for Viewpoint #8, the Hybrid AUI is most productive and efficient.

**Viewpoint #9: Subjects = Manual AUI Task Completion Time and Treatments = Age Groups:** Table 25 shows a summary of this viewpoint. Table 26 shows the results from one-way ANOVA for this viewpoint.

**Table 25:** Data summary—Viewpoint #9.

Treatment	Subject Count	Mean	Variance
18–30	24	4.32	0.59
31–44	18	5.94	0.54
45–70	18	6.9	0.43

**Table 26:** One-way ANOVA—Viewpoint #9.

*Source of Variation*	*SS*	*df*	*MS*	*F*	*P-value*	*F crit*
Treatments (AUIs)	69.31	2	34.65	60.27	8.65E-15	3.15
Subjects	32.77	57	0.57			
Total	102.08	59				

As can be seen in Table 26, there is a significant effect of age on participants’ task completion time for the Manual AUI, F(2, 57) = 3.15, p < 0.05. Therefore, the null hypothesis is refuted, and the alternative hypothesis is supported. Using Tukey’s HSD test the value of HSD is calculated: HSD = 0.58. As the absolute difference between the means of all the age groups greater than the HSD, all the age groups are statistically different in terms of their task completion speed, thus the age shows to have an impact on the usage of Manual AUI.

**Viewpoint #10: Subjects = Automatic AUI Task Completion Time and Treatments = Age Groups:** Table 27 shows a summary of this viewpoint. Table 28 shows the results from one-way ANOVA for this viewpoint.

**Table 27:** Data summary—Viewpoint #10.

Treatment	Subject Count	Mean	Variance
18–30	24	7.4	1.33
31–44	18	8.1	1.26
45–70	18	8.26	2.20

**Table 28:** One-way ANOVA—Viewpoint #10.

*Source of Variation*	*SS*	*df*	*MS*	*F*	*P-value*	*F crit*
Treatments (AUIs)	9.12	2	4.56	21.02	1.44E-07	3.15
Subjects	12.37	57	0.21			
Total	21.50	59				

As can be seen in Table 28, there is a significant effect of age on participants’ task completion time for the Automatic AUI, F(2, 57) = 3.15, p < 0.05. Therefore, the null hypothesis is refuted, and the alternative hypothesis is supported. Using Tukey’s HSD test the value of HSD is calculated: HSD = 0.36. The absolute difference between the means of age groups 31–44 and 45–70 is less than the HSD therefore these two groups are not statistically different. However, age group 18–30 is statistically different from the other two age groups. Thus, the age group 18–30 found the Automatic AUI to be more productive and efficient compared to the other age groups.

**Viewpoint #11: Subjects = Hybrid AUI Task Completion Time and Treatments = Age Groups:** Table 29 shows a summary of this viewpoint. Table 30 shows the results from one-way ANOVA for this viewpoint.

**Table 29:** Data summary—Viewpoint #11.

Treatment	Subject Count	Mean	Variance
18–30	24	3.92	0.41
31–44	18	3.95	0.28
45–70	18	3.96	0.16

**Table 30:** One-way ANOVA—Viewpoint #11.

*Source of Variation*	*SS*	*df*	*MS*	*F*	*P-value*	*F crit*
Treatments (AUIs)	0.01	2	8.31E-3	0.02	0.97	3.15
Subjects	18.76	57	0.33			
Total	18.78	59				

As can be seen in Table 30, there is no statistically significant effect of age on participants’ task completion time for the Hybrid AUI, F(2, 57) = 3.15, p < 0.05. Therefore, we cannot refute the null hypothesis. All age groups are shown to perform with a similar level of productivity and efficiency when using the Hybrid AUI.

### Questionnaire results

The survey was done at the end of the test. For each UI, the participants answered questions about each task, ranking the difficulty level from “Very Easy” to “Very Difficult”. Afterward, the scoring system shown in Table 31 was used to evaluate the answers.

**Table 31:** Scoring system for questions about difficulty.

Very Easy	Easy	Neutral	Difficult	Very Difficult
+2	+1	0	-1	-2

**Fig 18:**
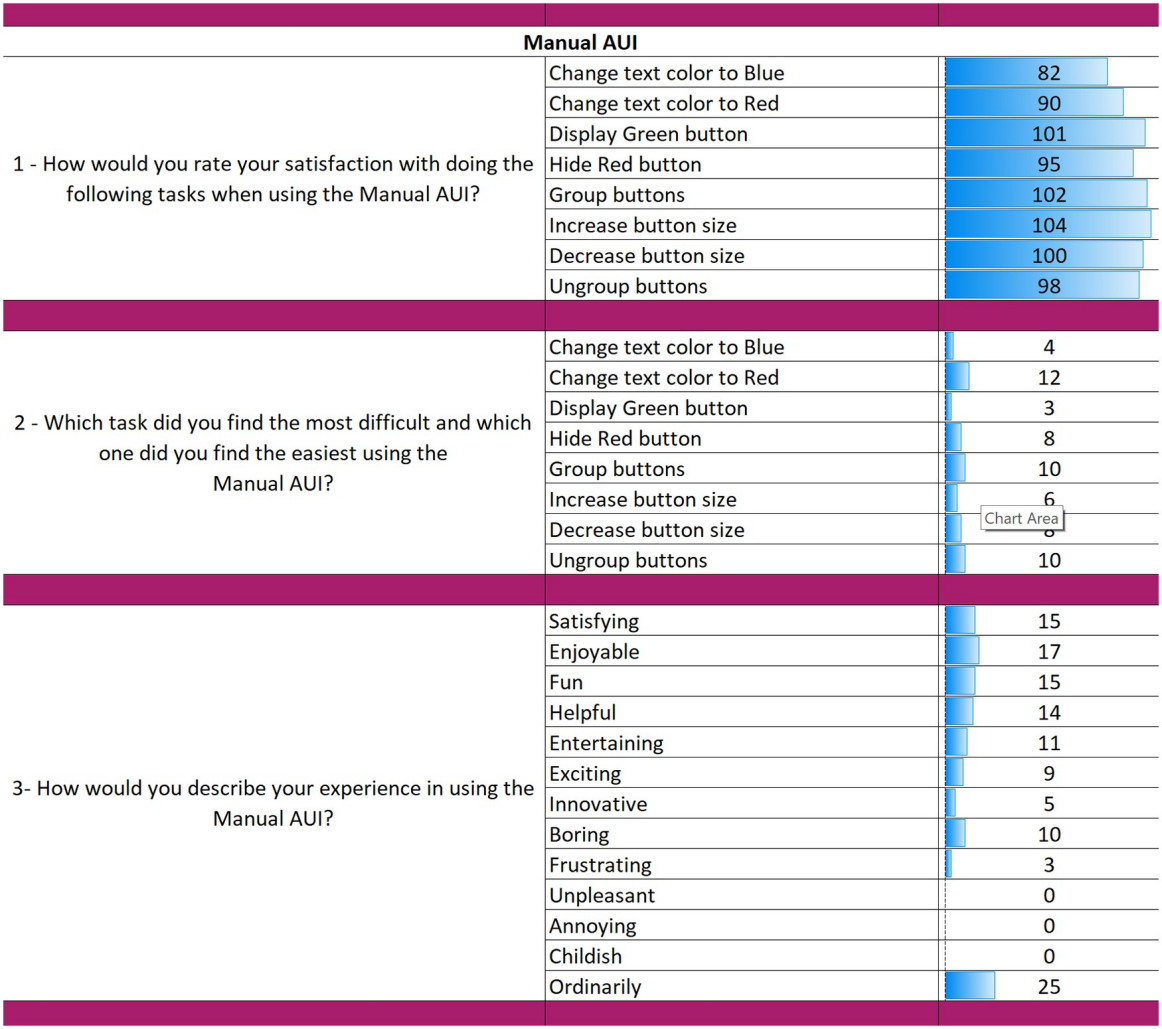
Questionnaire results for the Manual AUI.

**Fig 19:**
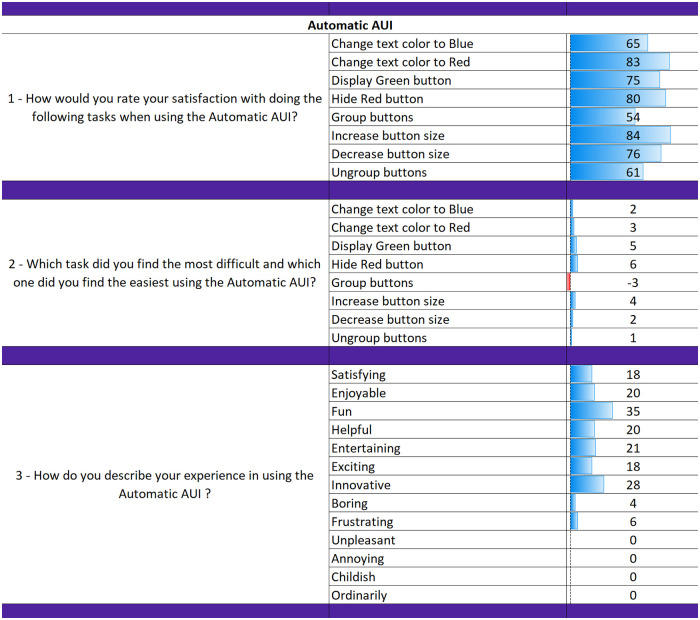
Questionnaire results for the Automatic AUI.

**Fig 20:**
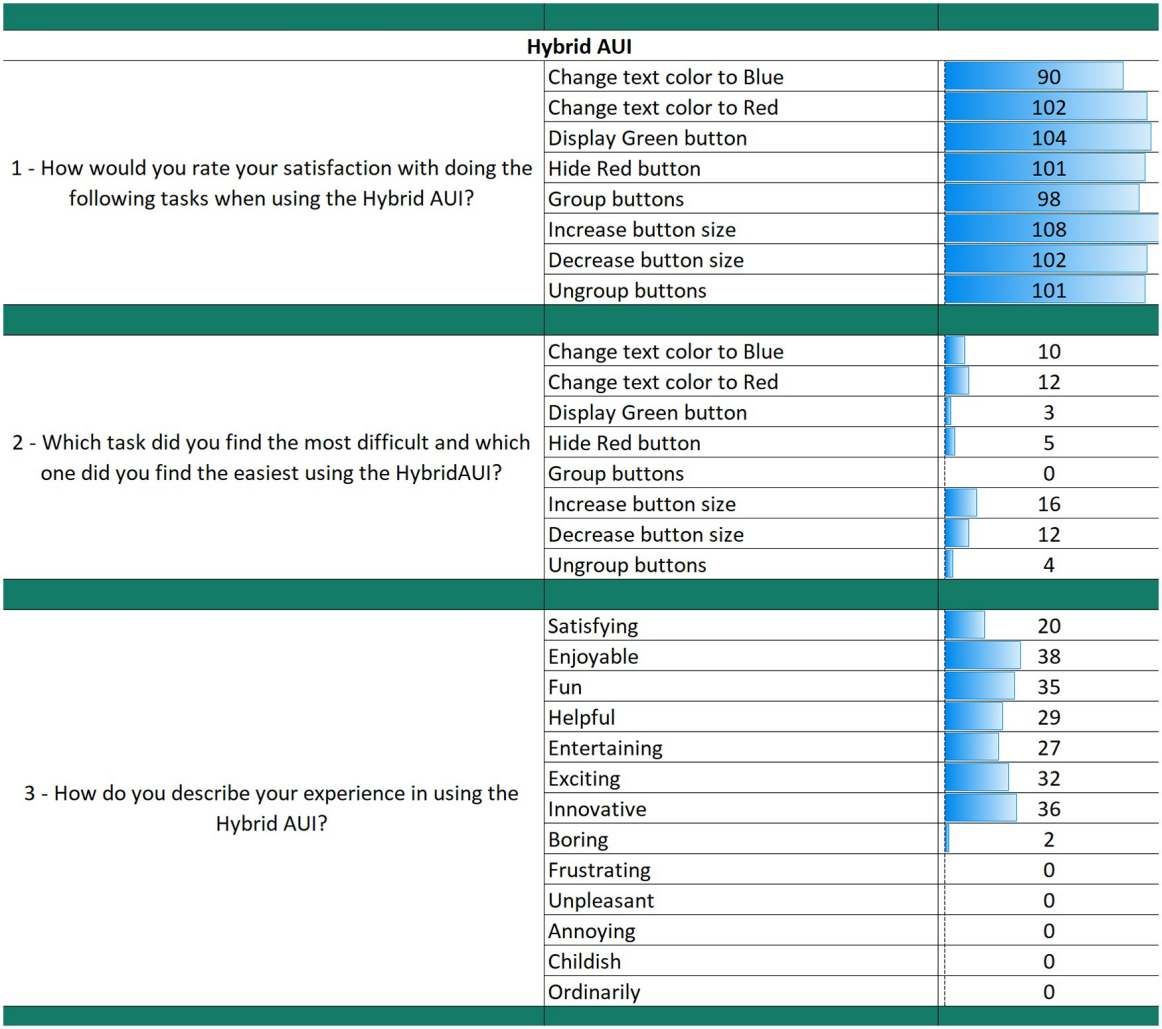
Questionnaire results for the Hybrid AUI.

**Fig 21:**
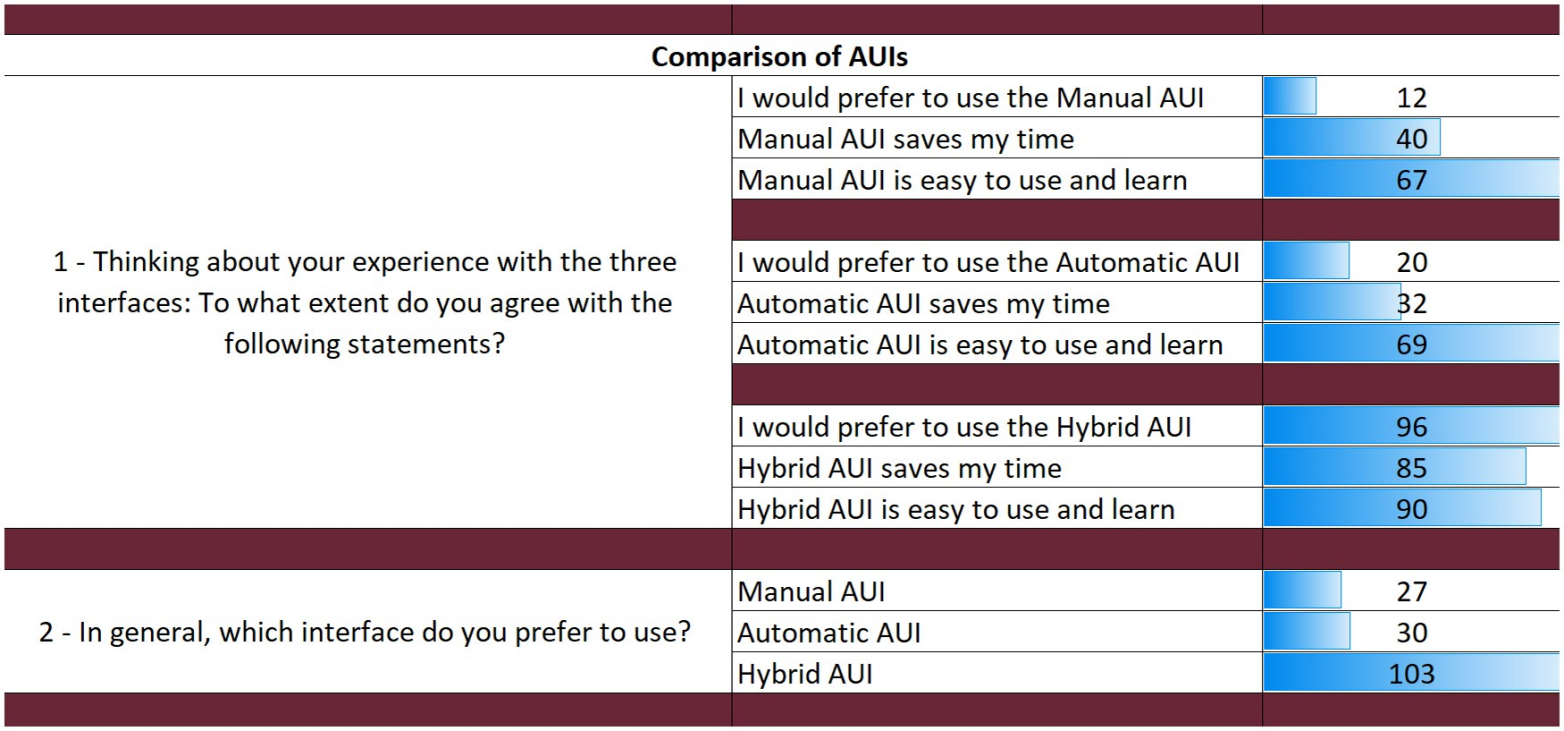
Questionnaire results for the comparison of three AUIs.

The results from the questionnaires for each AUI can be seen in Figs 18, 19 and 20. The final part of the questionnaire consists of a comparison between the three interfaces from the participants’ perspective. The results of the final part are shown in Fig 21.

### Qualitative feedback

As participants were performing different tasks, a range of reactions was observed. A semi-structured interview was conducted to explore theses reactions. From the answers of the participants, the following conclusions were drawn:

1. Participants greatly prefer the Hybrid AUI mainly because it provides immediate help through their emotional state but also because it gives them control to either adapt the interface manually or automatically.
2. Participants enjoyed the availability of speech commands to perform the different adaptation actions especially because it does not require manual interaction which reduces their effort.
3. Participants did not adhere to the concept of a fully automatic adaptation system available in the Automatic AUI mainly because it quickly becomes frustrating to be limited in terms of interaction.
4. Some participants found it interesting and comforting that the interface is able to advise them when they are feeling tired.

**Fig 22:**
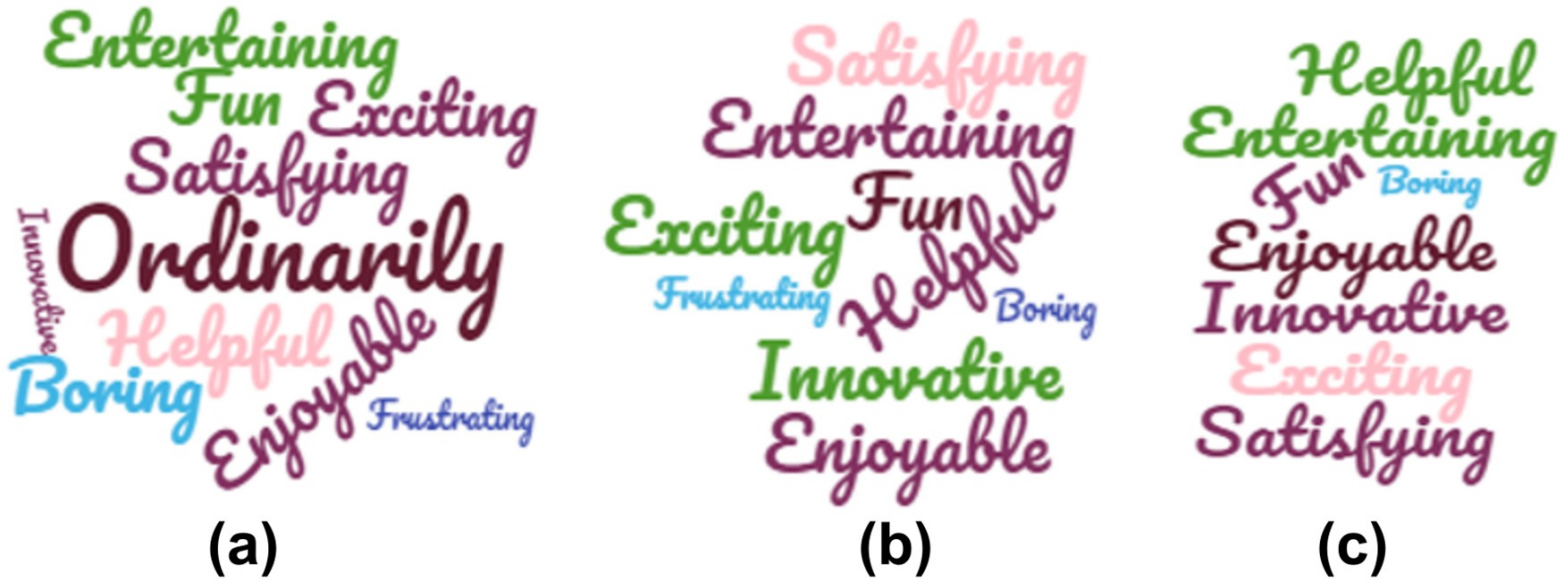
Keywords used by the participants to describe: (a) Manual AUI, (b) Automatic AUI, and (c) Hybrid AUI.

The participants were also asked to describe the different AUIs using different keywords. The results can be seen in Fig 22.

## Discussion

As can be seen in the previous section, a thorough usability and UX evaluation for all AUIs was performed using both quantitative and qualitative metrics. Based on the quantitative and qualitative data the following conclusions were draw about the three AUIs:

1. Manual AUI is the prevalent paradigm and all the users are extremely familiar with it. It has an element of personal control that an automatic adaptive system takes over. Many users prefer a degree of control when it comes to manipulating the UI because the feedback is immediate and there is no need to second guess the intention of the system.
2. Automatic AUI works silently in the background. It is supposed to be transparent because the UI space is precious, and one cannot add dynamic feedback based on the emotions on the screen. Microsoft tried with virtual assistants many times before that take the screen space, e.g. Clippy, but all of them were quickly discarded because users do not prefer an active agent on top of the screen. Giving control to a silent agent in the background to handle the adaptations causes frustrations to the user because he/she needs to second guess the intention of the system based on their emotion all the time. For example, it is possible for the user to not display any emotions despite frustrations. Therefore, only relying on the emotions to derive the adaptation will not work in this situation.
3. As shown by the results, a Hybrid AUI integrates the best of both worlds. The user always has a sense of control, when it comes to modifying the UI, on the other hand, the automatic adaptation system works automatically and supports the user even before the frustration with the manual system creeps in. The overwhelming feedback from the users show that they greatly prefer the hybrid system because based on their emotions it immediately offered them help to modify the UI. It was their choice to do it manually or automatically. Using the speech to quickly adapt the interface was much preferred. The automatic adaptation and the active response of the system to help the user was greatly appreciated and resulted in a much positive UX and usability score. Especially, as shown by the data analysis, the Hybrid AUI is the superior of all adaptive user interfaces as it is equivalent to all other AUIs, but it is better in terms of productivity and efficiency in most of the explored scenarios.
4. The data analysis identified statistical differences in terms of productivity and efficiency independently for each gender between the three AUIs, with Hybrid AUI being most productive and efficient.
5. The data analysis did not identify any statistical differences in terms of the usability parameters for any AUI due to gender differences.
6. The data analysis identified that the Manual and Hybrid AUI are statistically similar in terms of productivity and efficiency for the age group 18–30, but for the other age groups Hybrid AUI was the most productive and efficient.
7. The data analysis identified a statistical difference in terms of task completion time for different age groups, with the 18–30 age group taking the least time.

The results clearly demonstrate the benefits of the proposed study and show that implementing a Hybrid AUI can lead to a better UI design. With the advent of deep learning and move towards smart systems, a Hybrid AUI can easily be integrated into any software system. New learning technologies can be incorporated to recognize the emotions of the person even more effectively and the automatic adaptation can, foremost, reliably recognize the exact cause of user frustration and act accordingly.

### Limitations

The proposed method and user study have a couple of limitation. Firstly, a heuristic-based method was used for emotion recognition. Even though the accuracy of the proposed emotion recognition method is very high, it can be replaced by a different and potentially better learning method. Another reason why a learning method was not employed for the emotion recognition was due to the fact that RGB-D facial databases from Kinect are not widely available, and the three available databases [41–43] did not provide very good results for facial emotion recognition. Similarly, for the posture recognition, even for the most recent works [26], the database is not available publicly and does not deal with the fatigue posture. In the end compared to the earlier methods, the proposed heuristics-based emotion recognition methods provide very high accuracy and their effectiveness was validated in the user study.

Another limitation is the user study here focuses on one task at a time and only incorporates negative emotions. In principle, a user can perform multiple tasks, and the frustration can arise from the compound results of those tasks. The study focuses on only one task for now because it allows the testing of three AUIs. The handling of compound tasks across multiple AUIs introduces additional variables in the testing that would have required a very high number of participants for an unbiased evaluation. Similarly, positive emotions can be used as a measure of user’s satisfaction with UI and UX, but such measurements another layer of variables and complexity. Nonetheless, compound tasks and the ways to integrate positive emotions in an AUI will be handled in future work.

Despite all the limitations, this work shows that it is possible to augment a UI with active adaptive elements. The results confirmed that a Hybrid AUI with elements of both manual and automatic adaptations results in better usability and UX compared to a Manual AUI.

## Conclusions and future work

This work presented the design, implementation, and analysis of Automatic, Manual and Hybrid AUIs for a desktop application. The Manual AUI allows us to adapt a UI to the user’s needs with a manual interaction. A new heuristic-based solution was presented that recognizes the emotions of a user through both facial expressions and body posture. The emotion recognition was used to create an Automatic AUI. The Automatic AUI automatically modifies the UI elements based on the task performed by the user and tries to mitigate user’s frustration with the system. Finally, a Hybrid AUI was implemented that combines the elements of both Automatic and Manual AUI. The adaptations were designed to minimize the Gulf of Execution and Gulf of Evaluation that arise during user interactions. The goal of an AUI is to have a minimal gulf between the execution and evaluation.

The usability and UX of each AUI were validated by performing a comprehensive user study. The user study is performed on 60 participants, each performing 8 tasks with all three AUIs. Both quantitative and qualitative data were collected from the user study. The quantitative data is in the form of task completion time and error rate. The qualitative data is in the form of a semi-structured questionnaire. The hypothesis evaluation compared all three AUIs in terms of their effectiveness, productivity, efficiency, and error safety. Results show that the hybrid adaptation improves usability in terms of productivity and efficiency. Additionally, the data was evaluated from the viewpoints of genders and age groups.

Furthermore, the results show that both Automatic and Hybrid AUIs result in a significantly positive UX compared to the Manual AUI. In the future, the work will be extended by improving the underlying emotion recognition method. Additionally, the extension of the user study by the incorporation of compound tasks for the AUI evaluation will be explored. Finally, the future work will explore the use of even more modalities for emotion recognition, e.g. speech and voice, body cues, eye tracking, complex gestures, heart rate, and other physiological responses.

## Supporting information

S1 Appendix Reference emotions for calibrations. (DOCX)

S2 Appendix Questionnaire. (DOCX)
