# Supplementary material for: Adaptive user interface design and analysis using emotion recognition through facial expressions and body posture from an RGB-D sensor
Source: PLoS One. 2020 Jul 16;15(7):e0235908. doi: 10.1371/journal.pone.0235908 (PMC7365406; doi:10.1371/journal.pone.0235908)
Supplement: S1 Appendix — (DOCX) [file pone.0235908.s001.docx]

S1 Appendix. REFERENCE EMOTIONS FOR CALIBRATIONS

**Anger**

**
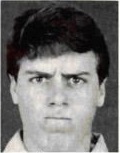

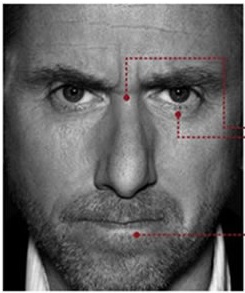

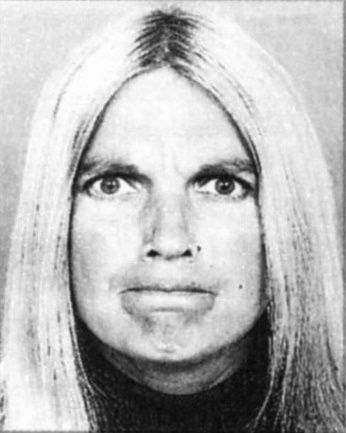
**

**Disgust**

**
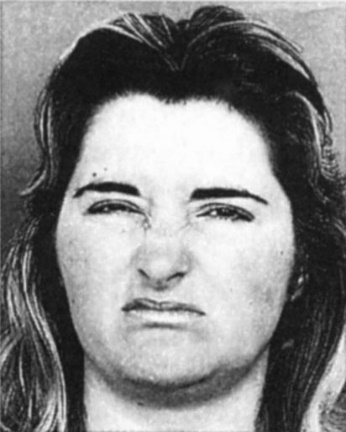

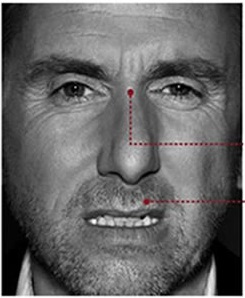

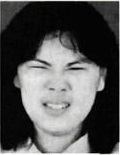
**

**Fear**

**
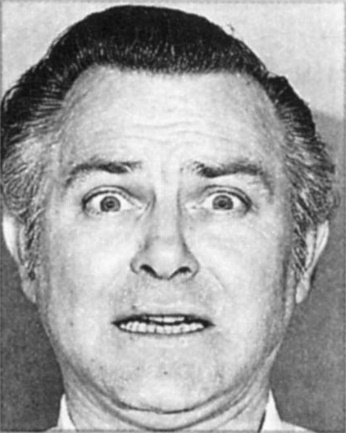

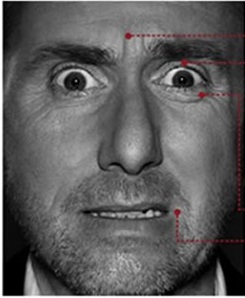

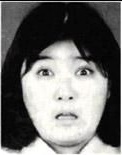
**

**Happiness**

**
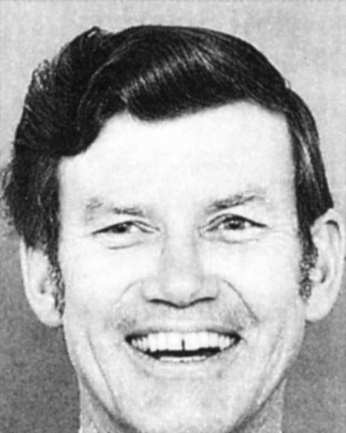

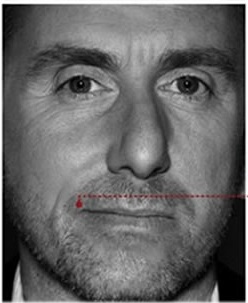

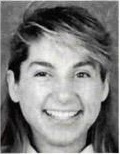
**

**Sadness**

**
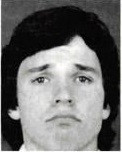

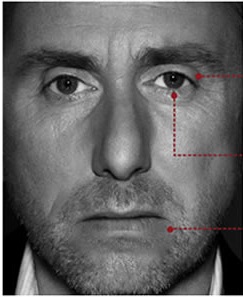

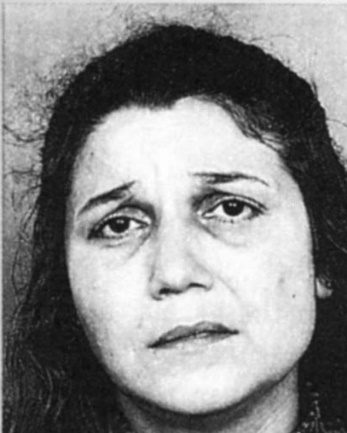
**

**Surprise**

**
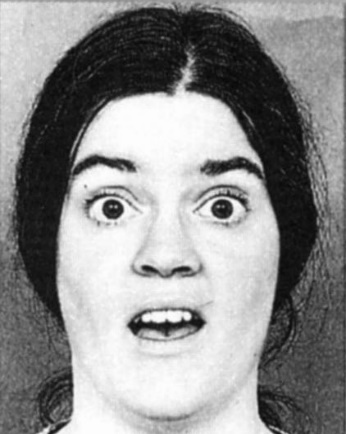

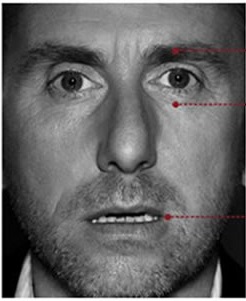

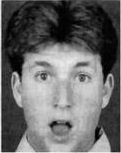
**
