# Supplementary material for: Adaptive user interface design and analysis using emotion recognition through facial expressions and body posture from an RGB-D sensor
Source: PLoS One. 2020 Jul 16;15(7):e0235908. doi: 10.1371/journal.pone.0235908 (PMC7365406; doi:10.1371/journal.pone.0235908)
Supplement: S2 Appendix — (DOCX) [file pone.0235908.s002.docx]

# **S2 Appendix Questionnaire**

| 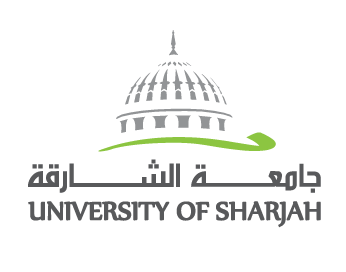 | | | | | |
| --- | --- | --- | --- | --- | --- |
| **Adaptive User Interface Design and Analysis using Emotion Recognition through Facial Expressions and Body Posture from an RGB-D Sensor** | | | | | |
| Thank you for agreeing to take part of this important survey measuring customer satisfaction. We are almost done with the evaluation. This questionnaire is designed to tell us how you feel about the different Adaptive User Interfaces you used today. Please check the answer that most clearly expresses how you feel about a statement. The answers you provide will be anonymous and kept confidential. Your cooperation is highly appreciated. | | | | | |
| 1. **General Questions:** | | | | | |
| 1. What is your gender?  - Female - Male | | | | | |
| 1. What is your age?  - 10 to 17 - 18 to 24 - 25 to 34 - 35 to 44 - 45 to 54 - 55 or older | | | | | |
| 1. Have you ever used this type of interfaces?  - Yes - No - If ‘Yes’, please specify what type of services/ applications do you use it for? | | | | | |
| 1. **Manual Adaptive User Interface:** | | | | | |
| 1. Please rate your satisfaction with doing the following tasks when using the Manual Adaptive User Interface? | | | | | |
|  | Very Easy | Easy | Neutral | Difficult | Very Difficult |
| Change text color to Blue |  |  |  |  |  |
| Change text color to Red |  |  |  |  |  |
| Display Green button |  |  |  |  |  |
| Hide Red button |  |  |  |  |  |
| Group buttons |  |  |  |  |  |
| Increase button size |  |  |  |  |  |
| Decrease button size |  |  |  |  |  |
| Ungroup buttons |  |  |  |  |  |
| 1. Which task did you find the most difficult and which one did you find the easiest using the Manual Adaptive User Interface?  - Change text color to Blue - Change text color to Red - Display Green button - Hide Red button - Group buttons - Increase button size - Decrease button size - Ungroup buttons | | | | | |
| 1. How do you describe your experience in using Manual Adaptive User Interface? (you can choose more than one answer)  - Satisfying - Enjoyable - Fun - Helpful - Entertaining - Exciting - Innovative - Boring - Frustrating - Unpleasant - Annoying - Childish - Ordinarily | | | | | |
| 1. **Automatic Adaptive User Interface:** | | | | | |
| 1. Please rate your satisfaction with doing the following tasks when using the Automatic Adaptive User Interface? | | | | | |
|  | Very Easy | Easy | Neutral | Difficult | Very Difficult |
| Change text color to Blue |  |  |  |  |  |
| Change text color to Red |  |  |  |  |  |
| Display Green button |  |  |  |  |  |
| Hide Red button |  |  |  |  |  |
| Group buttons |  |  |  |  |  |
| Increase button size |  |  |  |  |  |
| Decrease button size |  |  |  |  |  |
| Ungroup buttons |  |  |  |  |  |
| 1. Which task did you find the most difficult and which one did you find the easiest using the Automatic Adaptive User Interface?  - Change text color to Blue - Change text color to Red - Display Green button - Hide Red button - Group buttons - Increase button size - Decrease button size - Ungroup buttons | | | | | |
| 1. How do you describe your experience in using Automatic Adaptive User Interface? (you can choose more than one answer)  - Satisfying - Enjoyable - Fun - Helpful - Entertaining - Exciting - Innovative - Boring - Frustrating - Unpleasant - Annoying - Childish - Ordinarily | | | | | |
| 1. Do you think that Automatic Adaptive User Interface needs any improvement?  - Yes - No - If ‘Yes’, please specify how | | | | | |
| 1. **Hybrid Adaptive User Interface:** | | | | | |
| 1. Please rate your satisfaction with doing the following tasks when using the Hybrid Adaptive User Interface? | | | | | |
|  | Very Easy | Easy | Neutral | Difficult | Very Difficult |
| Change text color to Blue |  |  |  |  |  |
| Change text color to Red |  |  |  |  |  |
| Display Green button |  |  |  |  |  |
| Hide Red button |  |  |  |  |  |
| Group buttons |  |  |  |  |  |
| Increase button size |  |  |  |  |  |
| Decrease button size |  |  |  |  |  |
| Ungroup buttons |  |  |  |  |  |
| 1. Which task did you find the most difficult and which one did you find the easiest using the Hybrid Adaptive User Interface?  - Change text color to Blue - Change text color to Red - Display Green button - Hide Red button - Group buttons - Increase button size - Decrease button size - Ungroup buttons | | | | | |
| 1. How do you describe your experience in using Hybrid Adaptive User Interface? (you can choose more than one answer)  - Satisfying - Enjoyable - Fun - Helpful - Entertaining - Exciting - Innovative - Boring - Frustrating - Unpleasant - Annoying - Childish - Ordinarily | | | | | |
| 1. Do you think that Hybrid Adaptive User Interface needs any improvement?  - Yes - No - If ‘Yes’, please specify how | | | | | |
| 1. Thinking about your experience with the three interfaces: To what extent do you agree with the following statements? | | | | | |
|  | Strongly Agree | Agree | Neutral | Disagree | Strongly Disagree |
| I would prefer to use the Manual Adaptive User Interface |  |  |  |  |  |
| Manual Adaptive User Interface based interface saves my time |  |  |  |  |  |
| Manual Adaptive User Interface is easy to use and learn |  |  |  |  |  |
|  |  |  |  |  |  |
| I would prefer to use the Automatic Adaptive User Interface |  |  |  |  |  |
| Automatic Adaptive User Interface based interface saves my time |  |  |  |  |  |
| Automatic Adaptive User Interface is easy to use and learn |  |  |  |  |  |
|  |  |  |  |  |  |
| I would prefer to use the Hybrid Adaptive User Interface |  |  |  |  |  |
| Hybrid Adaptive User Interface based interface saves my time |  |  |  |  |  |
| Hybrid Adaptive User Interface is easy to use and learn |  |  |  |  |  |
| 1. In general, which interface do you prefer to use?  - Manual Adaptive User Interface - Automatic Adaptive User Interface - Hybrid Adaptive User Interface | | | | | |
